# Analysis of the Plasmid-Based *ts*-Mutant Δ*fabA/pTS-fabA* Reveals Its Lethality under Aerobic Growth Conditions That Is Suppressed by Mild Overexpression of *desA* at a Restrictive Temperature in Pseudomonas aeruginosa

**DOI:** 10.1128/spectrum.01338-23

**Published:** 2023-05-16

**Authors:** Liyan Tian, Zhili Yang, Jianxin Wang, Jianhua Liu

**Affiliations:** a Systems Biology, School of Marine Science and Technology, Zhejiang Ocean University, Zhoushan, Zhejiang, China; University of Manitoba

**Keywords:** conditional allele, essential gene, *fabA*, overexpression, suicide plasmid, suppressor, *Pseudomonas aeruginosa*

## Abstract

It is uncertain whether *PA1610|fabA* is essential or dispensable for growth on LB-agar plates under aerobic conditions in Pseudomonas aeruginosa PAO1. To examine its essentiality, we disrupted *fabA* in the presence of a native promoter-controlled complementary copy on *ts*-plasmid. In this analysis, we showed that the plasmid-based *ts*-mutant Δ*fabA*/*pTS-fabA* failed to grow at a restrictive temperature, consistent with the observation by Hoang and Schweizer (T. T. Hoang, H. P. Schweizer, J Bacteriol 179:5326–5332, 1997, https://doi.org/10.1128/jb.179.17.5326-5332.1997), and expanded on this by showing that Δ*fabA* exhibited curved cell morphology. On the other hand, strong induction of *fabA-OE* or *PA3645|fabZ-OE* impeded the growth of cells displaying oval morphology. Suppressor analysis revealed a mutant *sup* gene that suppressed a growth defect but not cell morphology of Δ*fabA*. Genome resequencing and transcriptomic profiling of *sup* identified *PA0286*|*desA*, whose promoter carried a single-nucleotide polymorphism (SNP), and transcription was significantly upregulated (level increase of >2-fold, *P* < 0.05). By integration of the SNP-bearing promoter-controlled *desA* gene into the chromosome of Δ*fabA*/*pTS-fabA*, we showed that the SNP is sufficient for Δ*fabA* to phenocopy the *sup* mutant. Furthermore, mild induction of the *araC*-P_BAD_-controlled *desA* gene but not *desB* rescued Δ*fabA*. These results validated that mild overexpression of *desA* fully suppressed the lethality but not the curved cell morphology of Δ*fabA*. Similarly, Zhu et al. (Zhu K, Choi K-H, Schweizer HP, Rock CO, Zhang Y-M, Mol Microbiol 60:260–273, 2006, https://doi.org/10.1111/j.1365-2958.2006.05088.x) showed that multicopy *desA* partially alleviated the slow growth phenotype of Δ*fabA*, the difference in which was that Δ*fabA* was viable. Taken together, our results demonstrate that *fabA* is essential for aerobic growth. We propose that the plasmid-based *ts*-allele is useful for exploring the genetic suppression interaction of essential genes of interest in P. aeruginosa.

**IMPORTANCE** Pseudomonas aeruginosa is an opportunistic pathogen whose multidrug resistance demands new drug development. Fatty acids are essential for viability, and essential genes are ideal drug targets. However, the growth defect of essential gene mutants can be suppressed. Suppressors tend to be accumulated during the construction of essential gene deletion mutants, hampering the genetic analysis. To circumvent this issue, we constructed a deletion allele of *fabA* in the presence of a native promoter-controlled complementary copy in the *ts*-plasmid. In this analysis, we showed that Δ*fabA*/*pTS-fabA* failed to grow at a restrictive temperature, supporting its essentiality. Suppressor analysis revealed *desA*, whose promoter carried a SNP and whose transcription was upregulated. We validated that both the SNP-bearing promoter-controlled and regulable P_BAD_ promoter-controlled *desA* suppressed the lethality of Δ*fabA*. Together, our results demonstrate that *fabA* is essential for aerobic growth. We propose that plasmid-based *ts*-alleles are suitable for genetic analysis of essential genes of interest.

## INTRODUCTION

Pseudomonas aeruginosa is a Gram-negative rod-shaped bacterium commonly found in soil and water. It is a major pathogen in the cystic fibrosis lung with high prevalence (1). Frequent appearance of drug-resistant strains leads to high mortality in immunocompromised patients (2). The World Health Organization has classified P. aeruginosa as an important pathogenic bacterium requiring research investment and new drug development (3).

Essential genes are suitable targets for the development of new drugs because disruption of essential genes causes microbial cell death (4, 5). Nevertheless, many essential genes are found to be suppressible (6), partly caused by spontaneously occurring suppressors during construction of essential gene deletion strains (6, 7). Spontaneous accumulation of suppressor mutations can be prevented by the presence of a complementary copy in the *ts*-plasmid during construction of essential gene deletion strains at permissive temperatures (8). On the other hand, plasmid-based conditional mutant strains are suitable for suppressor screening at a restrictive temperature (8, 9).

Fatty acids are essential for cell viability (10). While human type I fatty acid synthesis (FASI) is carried out by a multifunctional single protein composed of distinct enzymatic domains, bacterial type II fatty acid synthesis (FASII) is accomplished by a series of individual enzymes, which makes bacterial type II fatty acids ideal targets for development of antibiotics (see 11 and 12 and references therein). PA1614|FabA is a bifunctional enzyme: it is a 3-hydroxyacyl-acyl carrier protein (ACP) dehydratase (EC 4.2.1.59) that catalyzes the formation of *trans*-2-enoyl-ACP from substrate β-hydroxyacyl-ACP, which is involved in saturated fatty acid synthesis in the FASII elongation cycle (13, 14); it is also a *trans*-2-decenoyl-ACP isomerase (EC 5.3.3.14) that catalyzes the reaction of a cis-3-decenoyl-ACP formation from a *trans*-3-decenoyl-ACP, which is involved in unsaturated fatty acid synthesis (14–16). PA3645|FabZ, another copy of 3-hydroxyacyl-ACP dehydratase (EC 4.2.1.59), is interchangeable with FabA in the cycles of fatty acid elongation up to 10 carbons (16). It is proposed that FabA is more active in the dehydration of β-hydroxydecanoyl-ACP for the formation of *cis*-3-decenoyl-ACP for unsaturated fatty acid synthesis (16), while FabZ is the major dehydratase in the elongation cycles of saturated fatty acid biosynthesis (16).

In Escherichia coli, the *fabA* gene is essential for growth on LB plates and auxotrophic for oleic acid (17–20). However, the essentiality of *fabA* in Pseudomonas aeruginosa is complex: it is essential for growth on LB and auxotrophic for oleic acid according to some studies (4, 21) but not essential for aerobic growth according to others (22). Reasons for this discrepancy are not clear. It could be caused by different backgrounds in laboratory strains or the spontaneous occurrence of suppressors during construction of essential gene deletion strains without protection by complementary copies.

To address this issue, we have carried out a three-step approach (8) to construct a plasmid-based *ts*-mutant strain, Δ*fabA*/*pTS-fabA*, that contains a Δ*fabA* allele on chromosome and a complementary copy of *fabA* in the *ts*-plasmid. We show that Δ*fabA*/*pTS-fabA* fails to grow at a restrictive temperature and exhibits a terminal phenotype of curved cell morphology under aerobic growth conditions. Strong induction of *fabA-OE* or *fabZ-OE* hampers the growth of cells that display oval cell morphology. Furthermore, spontaneous suppressor screening identified a *sup* mutant that rescued the growth defect but not morphology of Δ*fabA*. Genome re-sequencing and transcriptomic profiling revealed *desA*, whose promoter bore a single nucleotide polymorphism (SNP) and whose transcription was upregulated. We validated that both the SNP-bearing promoter-controlled and regulable *araC*-P_BAD_ promoter-controlled *desA* could rescue the Δ*fabA* growth defect. Taken together, our results show that *fabA* plays an essential role in the regulation of cell morphology in an expression level-dependent manner in P. aeruginosa.

## RESULTS

### The *fabA* gene is essential for growth on LB agar plates under aerobic conditions.

**FIG 1:**
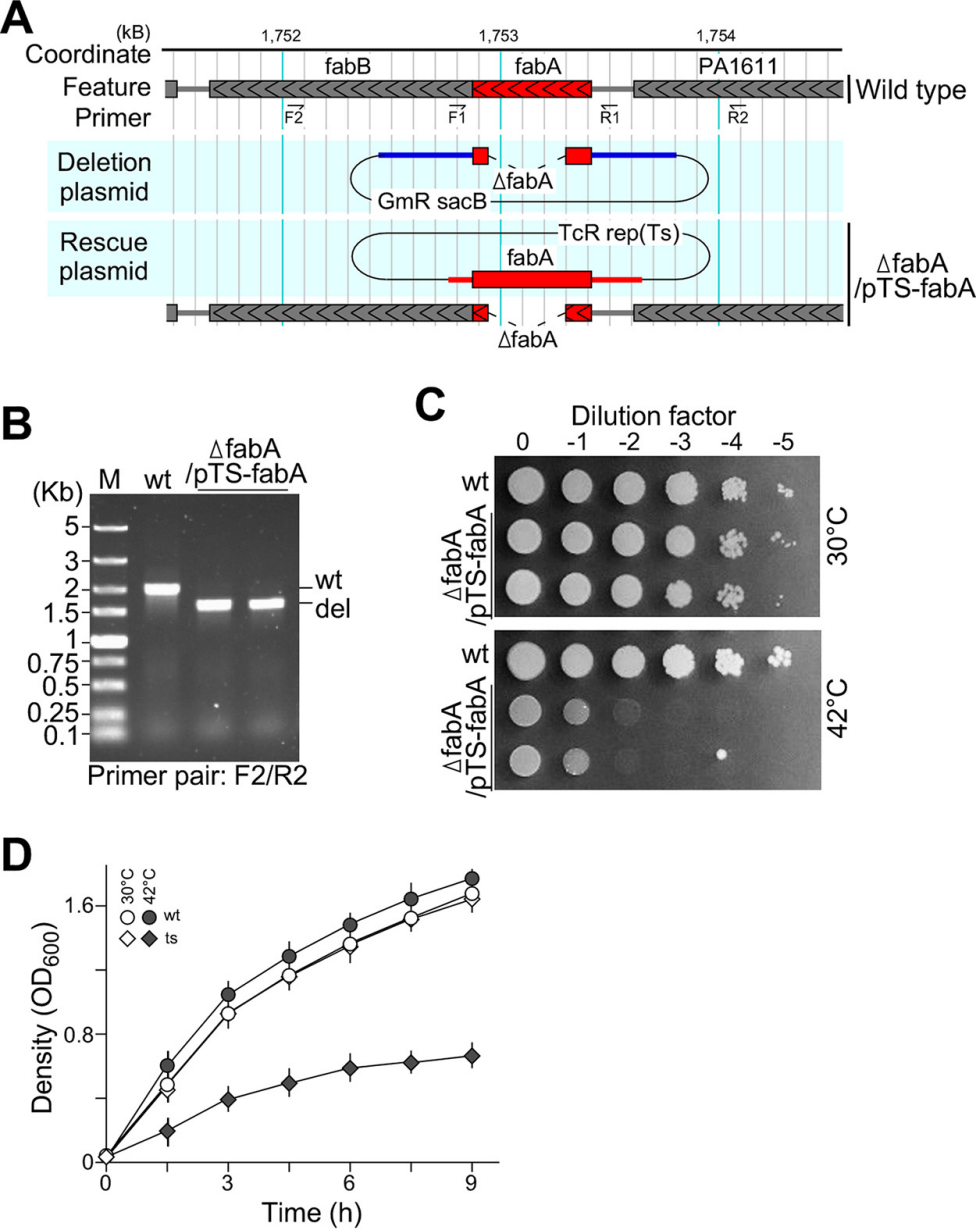
Δ*fabA*/*pTS-fabA* fails to grow on LB under aerobic conditions at a restrictive temperature. (A) Physical map of the *fabA* deletion allele cassette (blue line) in the deletion plasmid and complementary sequences (red line) in the rescue cassette. Δ*fabA*/*pTS-fabA* contains a deletion allele, Δ*fabA*, on the chromosome and a native promoter-controlled complementary copy, *fabA*, on the *ts*-sensitive plasmid. (B) PCR assay for *fabA* alleles. By using F2-R2 primer pairs located outside the *fabA* complementary sequences on the rescue cassette, the chromosomal deletion allele of *fabA* is found in Δ*fabA*/*pTS-fabA* isolates. (C) Spot-plating assay for Δ*fabA*/*pTS-fabA* growth under aerobic conditions. A series of 10-fold serially diluted wild-type and mutant cells were spotted on an LB plate and incubated at 30°C and 42°C overnight. (D) Growth curves of the second subcultures. The *x* and *y* axes show the time (h) and cell density (OD_600_), respectively.

Construction of essential gene deletion strains was often associated with the occurrence of spontaneous suppressors in bacteria (7). To prevent suppressors from occurring, we deleted the *fabA* chromosomal copy in the presence of a complementary copy in the *ts*-plasmid. That is, by using a three-step protocol (8), the chromosomal deletion Δ*fabA* allele was created under the protection of a native promoter-controlled *fabA* in the *ts*-plasmid pTS-*fabA* (Fig. 1A) (see Materials and Methods). The chromosomal deletion allele Δ*fabA* in this *ts*-plasmid-based mutant strain, Δ*fabA*/*pTS-fabA* was confirmed by PCR assay using primer pairs F2 and R2, whose priming sites were absent in the complementary copy of the rescue plasmid pTS-*fabA* (Fig. 1B). A spot-plating assay indicated that the growth of Δ*fabA*/*pTS-fabA* was impeded at a restrictive temperature of 42°C on an LB plate under aerobic conditions compared to that of wild type, while the growth of Δ*fabA*/*pTS-fabA* was nearly identical to that of the wild type at a permissive temperature of 30°C (Fig. 1C). This result indicated that *fabA* is essential for growth on LB agar under aerobic conditions, consistent with the observation by Hoang and Schweizer (21). In contrast, Zhu et al. showed that Δ*fabA* was viable but exhibited a slow growth phenotype under aerobic condition (22). This discrepancy could be a result of different substrain backgrounds (23) or could, alternatively, be caused by spontaneous suppressor accumulations during construction of the deletion strain (6, 7).

Depletion of the multicopy *ts*-plasmid in mutant cells would take several generations at a restrictive temperature. We found that the growth curve of Δ*fabA*/*pTS-fabA* reached a critical point at 4.5 h with an optical density at 600 nm (OD_600_) of ~1.2 on average at the onset of the stationary phase after a temperature shift (see Materials and Methods). That is, prior to the critical point, cell growth resembled that of the wild type. In contrast, after that point, the growth rate slowed down compared to that of wild type, suggesting the loss of complementary plasmids in mutant cells (see Fig. S1A and B in the supplemental material). However, it was less efficient for slow-growing mutant cells to develop the phenotype. To resolve this issue, we devised a consecutive subculture method to allow cells reducing the plasmid copy number in the first subculture and continuing phenotype development in the second subculture (Fig. S1C and D). We found that copy numbers of the complementary plasmid were higher than those of the chromosome in the Δ*fabA*/*pTS-fabA* strain at the beginning of the second subculture based on the reverse transcription-quantitative PCR (qRT-PCR) analysis comparing plasmid-specific and chromosome-specific sequences (see Materials and Methods). Hence, we used the growth curve of the second subculture to show the growth defect of Δ*fabA*/*pTS-fabA* at a restrictive temperature (Fig. 1D).

### The Δ*fabA*/*pTS-fabA* mutant exhibits curved cell morphology at a restrictive temperature.

FabA, a bifunctional enzyme of 3-hydroxyacyl-ACP dehydratase (EC 4.2.1.59) and *trans*-2-decenoyl-ACP isomerase (EC 5.3.3.14), was involved in the synthesis of both saturated and unsaturated fatty acids (13–16, 21). To investigate the effect of FabA depletion on cell morphology, we examined the terminal phenotype of Δ*fabA*/*pTS-fabA* at a restrictive temperature. For this reason, both the Δ*fabA*/*pTS-fabA* mutant and wild-type strains were subjected to a temperature shift from 30°C to 42°C. Samples of the second subculture at 0 h, 3 h, and 6 h at a restrictive temperature were fixed and stained with Nile red prior to fluorescence microscopic analysis (see Materials and Methods).

**FIG 2:**
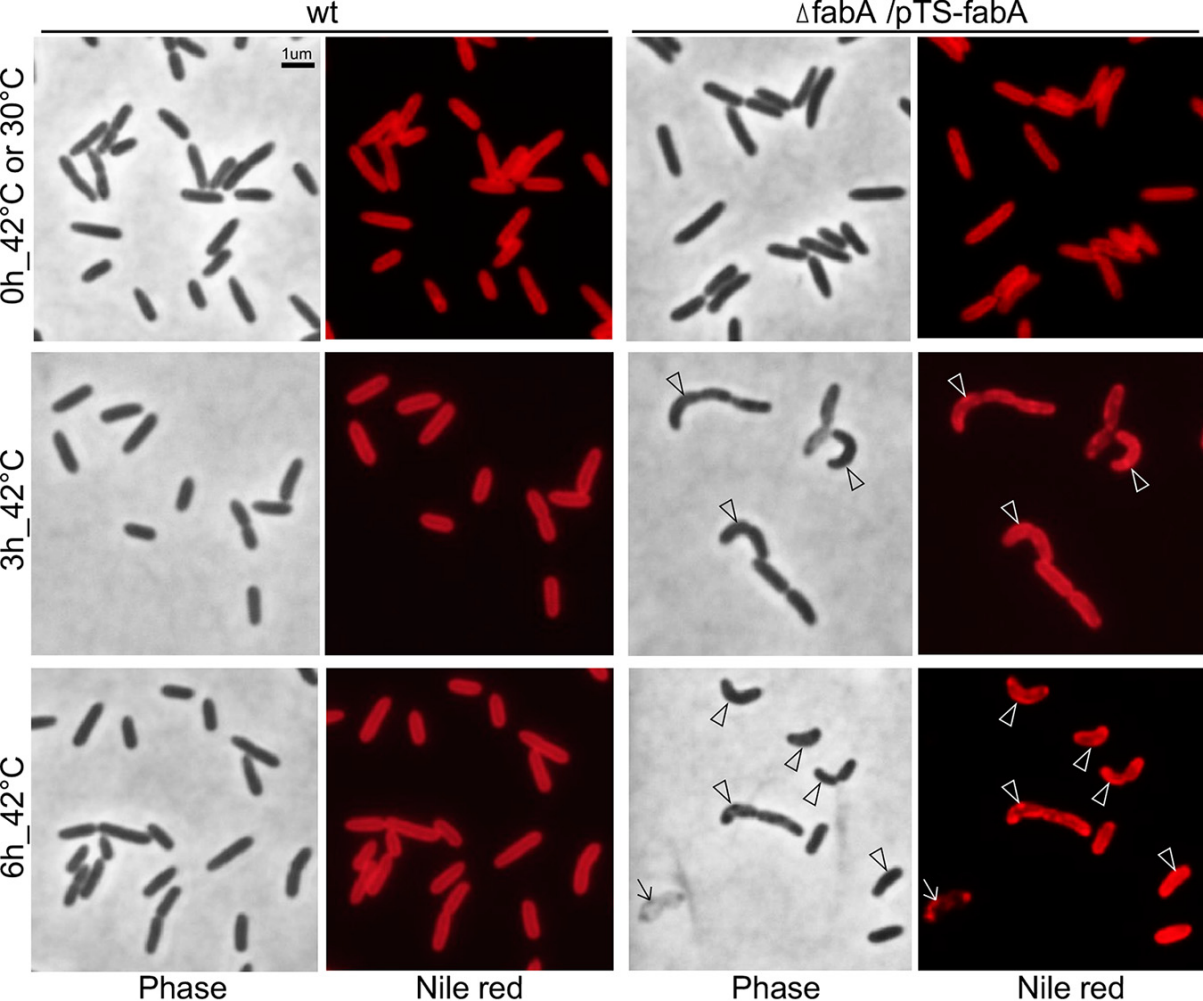
Δ*fabA*/*pTS-fabA* exhibits curved cell morphology at a restrictive temperature under aerobic conditions. The cells were examined prior to the temperature shift from 30°C to 42°C starting the first subculture or at 0 h, 3 h, and 6 h after the second subculture was started at 42°C. Arrowheads indicate the curvature of the curved cells. Arrows indicate the ghost cell.

Upon starting the second subculture (0 h), Δ*fabA*/*pTS-fabA* exhibited a wild-type-like rod-shaped cell morphology (Fig. 2, top row). On the other hand, 3 h after growth, Δ*fabA*/*pTS-fabA* mutant cells exhibited curved morphology (Fig. 2, middle row, see arrowheads). Strong Nile red fluorescence signals were found at the curvature of curved cells. It was proposed that new cell wall materials were evenly inserted along the cylindrical part of the rod-shaped bacterial cell during polarized growth (24). The curved cell of Δ*fabA*/*pTS-fabA* could be a result of uneven distribution of the newly synthesized cell wall materials to the cylindrical portion. After growth for 6 h in the second subculture, in addition to the curved cells, ghost cells or lysed cells started to appear (Fig. 2, bottom row, see arrows). These results indicated that cells lacking FabA function failed to maintain the rod-shaped cell morphology.

Though the *ts*-plasmid-based mutant would take a number of generations to for phenotypes in liquid culture to be observed, it is worth noting that the *ts*-plasmid-based conditional allele could be constructed for most, if not all, essential genes, while the point mutation-based conditional allele would not. Additionally, the time required for mutants to develop phenotypes in liquid culture was insufficient for spontaneous suppressors to populate the culture. Hence, the *ts*-plasmid-based mutant strains were suitable for systematic deletion analysis of essential genes in P. aeruginosa.

### The Δ*fabA*/pTS-*fabA* mutant strain exhibits a significantly decreased level of palmitoleic acid at restrictive a temperature compared to that of the wild type.

**FIG 3:**
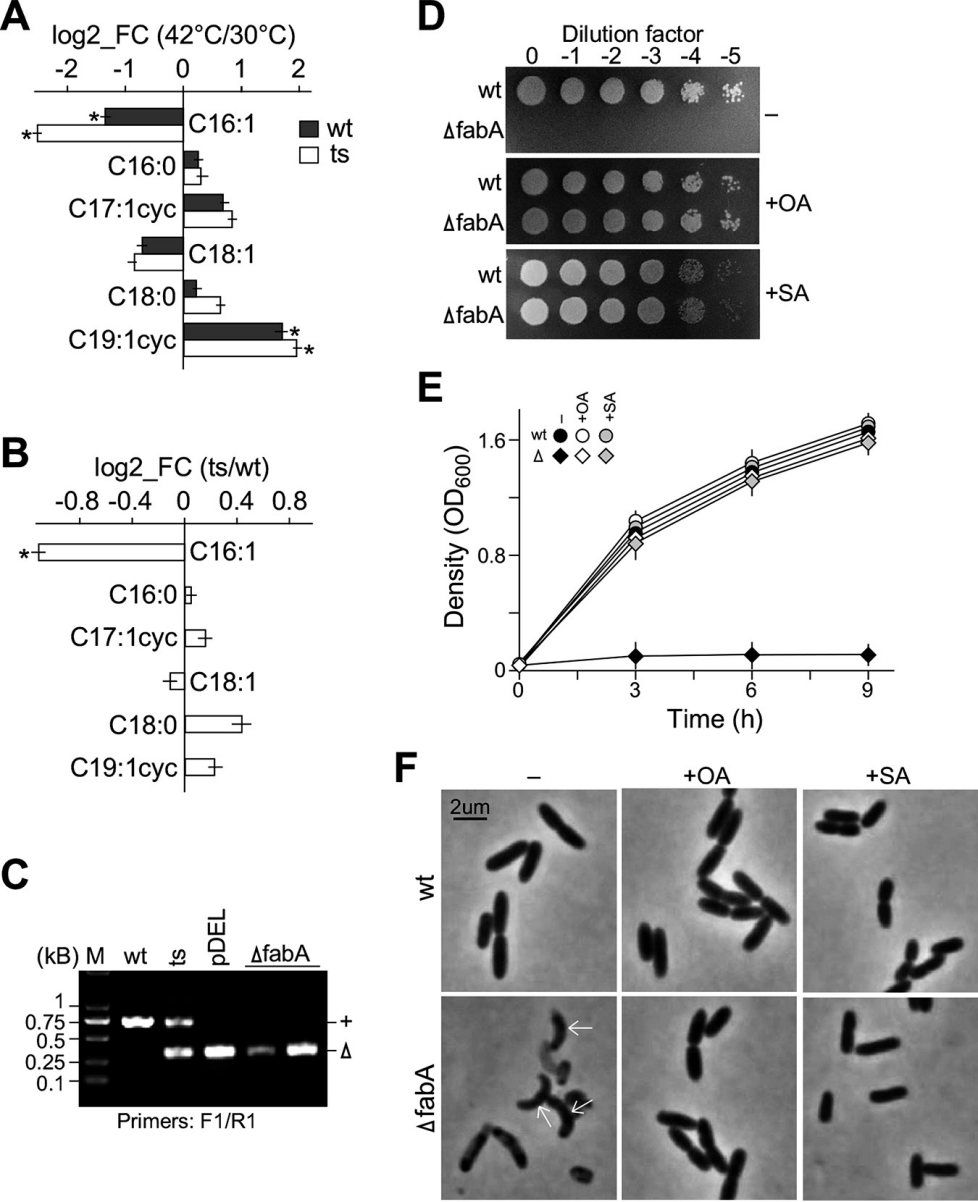
Δ*fabA*/*pTS-fabA* exhibits a decreased palmitoleic acid level and is auxotrophic for oleic acid and stearic acid at a restrictive temperature. (A) Level changes of various fatty acids after the temperature shift to 42°C compared to that at 30°C in Δ*fabA*/*pTS-fabA* (ts) and wild-type (wt) cells. The *x* and *y* axes show the ratio (levels at 42°C over 30°C) and species of fatty acids indicated, respectively. *, *P* < 0.05; *n* = 3. (B) Difference in fatty acid (FA) level changes between Δ*fabA*/*pTS-fabA* and the wild type. The *x* and *y* axes indicate the ratio between Δ*fabA*/*pTS-fabA* and the wild type, respectively, and various fatty acids. (C) Gel image showing the isolation of Δ*fabA* from Δ*fabA/pTS*-*fabA* in medium supplemented with oleic acid at a restrictive temperature. (D) Spot-plating assay showing Δ*fabA* that failed to grow on medium without supplementation of oleic acid or stearic acid under aerobic conditions. (E) Growth curve of Δ*fabA* and wild-type strains in LB medium with or without oleic acid or stearic acid supplementation. The *x* and *y* axes indicate time (h) and cell density (OD_600_), respectively. (F) Microscopic images showing Δ*fabA* and wild-type cells in LB medium supplemented with and without oleic acid or stearic acid. Arrows indicate the curvature of the cells.

To investigate the effect of *fabA* disruption on fatty acid biosynthesis, we determined the relative level of various fatty acids in the Δ*fabA*/*pTS-fabA* mutant and wild-type cells at 30°C and 42°C based on the gas chromatography-mass spectrometry (GC-MS) analysis after transesterification. In this analysis, cellular lipids were extracted using chloroform and methanol solution (2:1 vol/vol) (25, 26). The resulting lipids were transesterified to generate fatty acid methyl esters (FAME) (27) (see Materials and Methods). Six major fatty acids were found in both the wild-type and Δ*fabA*/*pTS-fabA* strains at 30°C and 42°C: palmitoleic acid (C_16:1_), palmitic acid (C_16:0_), cyclopropaneoctanoic acid 2-hexyl (C_17:lcyc_), oleic acid (C_18:1_), stearic acid (C_18:0_), and cyclopropaneactanoic acid 2-octyl (C_19:lcyc_) (Fig. S2). Upon a temperature shift to 42°C, we found two fatty acid species, C_16:1_ and C_19:lcyc_, whose level was significantly decreased and increased in both the wild-type and Δ*fabA*/*pTS-fabA* strains, respectively. Upon the temperature increase, the level of C_19:lcyc_ in Δ*fabA*/*pTS-fabA* was increased by 3.86-fold (i.e., average of 4.40-fold at 30°C versus 17.07-fold at 42°C; *P* < 0.05, *n* = 3), which was close to 3.27-fold in the wild type (Fig. 3A and B, Table S1). On the other hand, after the temperature increase to 42°C, the level of C_16:1_ was decreased to 18% of the initial level at 30°C (i.e., average of 13.05 arbitrary unit at 30°C versus 2.29 arbitrary unit at 42°C; *P* < 0.05, *n* = 3) in Δ*fabA*/*pTS-fabA*, which decreased by greater than 2-fold compared to that of wild type (i.e., 18% in Δ*fabA*/*pTS-fabA* versus 38% in the wild type; *P* < 0.05, *n* = 3). These results suggested that C_16:1_ biosynthesis was reduced in Δ*fabA*/*pTS-fabA* at a restrictive temperature.

It was shown that the *fabA^−^* mutant was auxotrophic for oleic acid and stearic acid (16, 21). Consistent with this, we isolated the Δ*fabA* mutant from Δ*fabA*/*pTS-fabA* at 42°C after depletion of the *ts*-rescue plasmid in medium supplemented with oleic acid or stearic acid (Fig. 3C). A spot-plating assay showed that Δ*fabA* failed to grow on an LB agar plate without oleic acid or stearic acid supplementation under aerobic conditions (Fig. 3D). Likewise, Δ*fabA* did not grow in LB liquid medium without oleic acid or stearic acid supplementation (Fig. 3E). Microscopic analysis indicated that after removal of oleic acid or stearic acid, Δ*fabA* exhibited curved morphology (Fig. 3F, see arrow), identical to that of Δ*fabA*/*pTS-fabA* at a restrictive temperature (see Fig. 2), supporting the notion that curved phenotype was attributed to the loss of FabA function.

### Cells fail to grow and exhibit oval morphology upon strong induction of *fabA-OE* or *fabZ-OE*.

**FIG 4:**
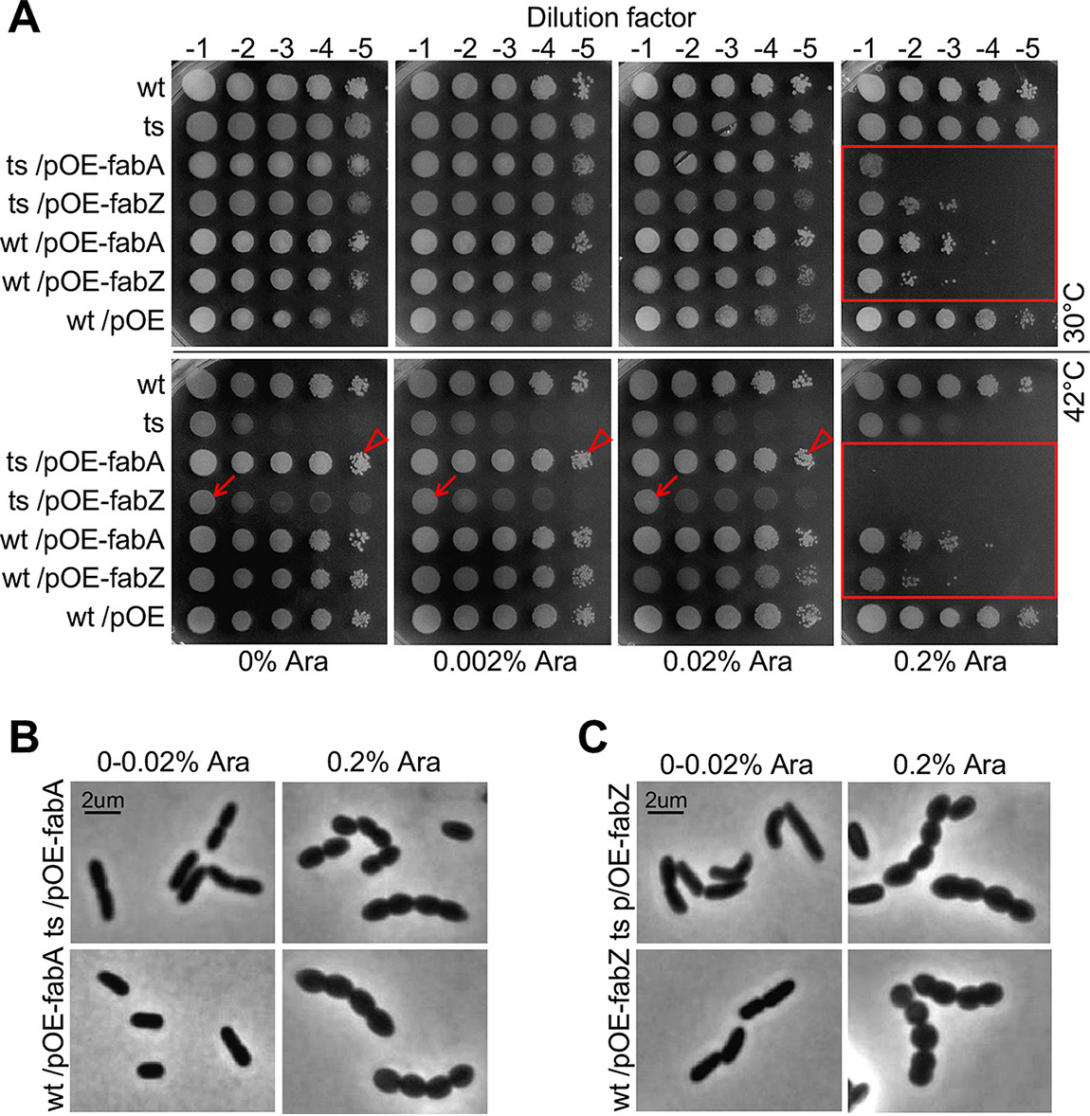
Strong induction of *fabA-OE* and *fabZ-OE* impedes cell growth. (A) Effect of *fabA-OE* and *fabZ-OE* with supplementation of arabinose at various concentrations. Arrowheads indicate the rescue of Δ*fabA*/*pTS-fabA* growth by mild induction of *fabA-OE* with 0 to 0.02% arabinose at a restrictive temperature. Arrows indicate no rescue with *fabZ-OE*. Rectangles indicate the slow growth by strong induction (0.2% arabinose) of *fabA-OE* and *fabZ-OE*. Wt/pOE served as the plasmid control. (B) Microscopic image showing different cell morphologies of Δ*fabA*/*pTS-fabA* and wild-type cells upon induction of *fabA-OE* with the various arabinose doses indicated. (C) Microscopic image showing different cell morphologies of Δ*fabA*/*pTS-fabA* and wild-type cells upon induction of *fabZ-OE* with the various arabinose doses indicated.

Another dehydratase, FabZ, was known to be present in P. aeruginosa (28). To examine whether overexpression of the *fabZ* gene, an isozyme of *fabA*, could rescue the growth defect of Δ*fabA*/*pTS-fabA* at a restrictive temperature, we prepared an overexpression *fabZ-OE* construct whose transcription was under the control of the arabinose-regulable P_BAD_ promoter (29) in the pBBR plasmid to generate pOE-*fabZ* (see Materials and Methods). As a control, the pOE-*fabA* plasmid was also constructed. We found that mild overexpression of *fabZ* with 0.002% and 0.02% arabinose did not rescue the growth defect of the Δ*fabA*/*pTS-fabA pOE-fabZ* strain at a restrictive temperature (Fig. 4A, see arrow). However, as a control, we found that no or mild overexpression of *fabA* rescued the growth defect of Δ*fabA/pTS-fabA pOE-fabA* strain at restrictive temperature (Fig. 4A, see arrowheads). This result suggested that FabZ did not share essential functions with FabA. In addition, *fabA* transcription at a level derived from leakiness or without induction of *fabA-OE* was sufficient for function.

Notably, we found that under strong induction with 0.2% arabinose, both *fabA-OE* and *fabZ-OE* impeded growth of the Δ*fabA*/*pTS-fabA* strain and the wild type (Fig. 4A, see rectangles). Morphological analysis showed that, under strong induction of *fabA-OE* or *fabZ-OE*, Δ*fabA*/*pTS-fabA* and wild-type cells exhibited a terminal phenotype of oval morphology at both 30°C and 42°C (Fig. 4B and C). This result suggested that a strong overexpression phenotype of *fabA-OE* and *fabZ-OE* was likely to be a result of overlapping function between FabA and FabZ.

### A suppressor, *sup*, restores cell growth but not cell morphology of Δ*fabA* at a restrictive temperature.

**FIG 5:**
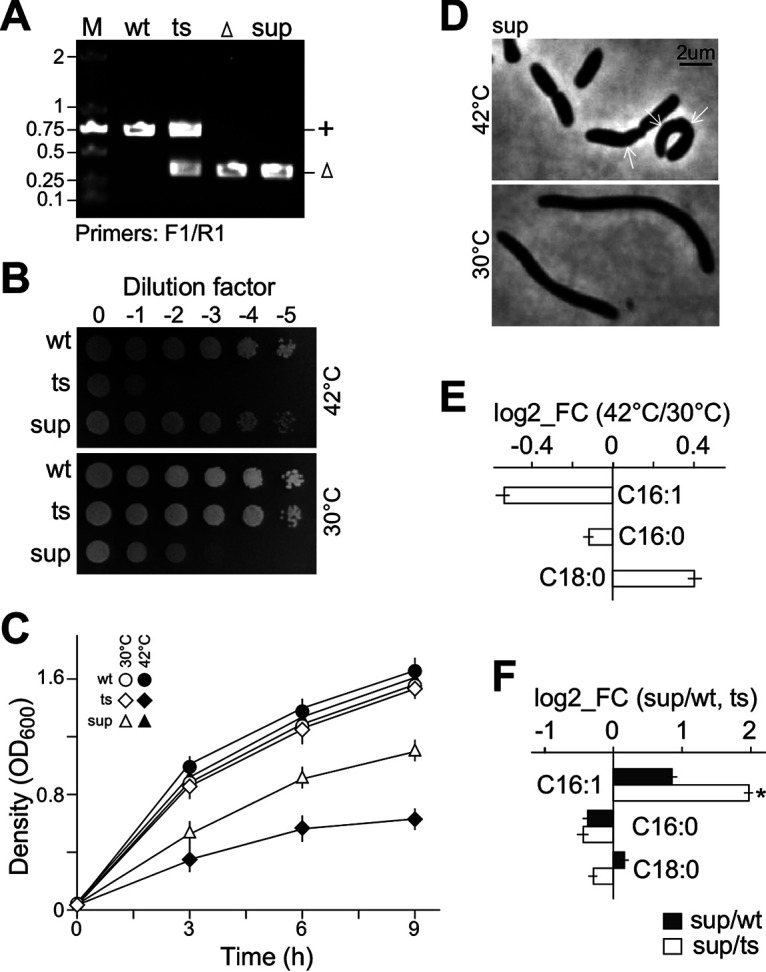
Isolation and phenotypic analysis of a suppressor (*sup*) of Δ*fabA*/*pTS-fabA* at a restrictive temperature. (A) A suppressor is identified from the suppressor screen. A gel image shows the *sup* strain containing only the Δ*fabA* allele. (B) Spot-plating assay of the *sup* strain. (C) Growth curve analysis of the wild type, Δ*fabA*/*pTS-fabA*, and *sup* at 30°C and 42°C. (D) Microscopic analysis of *sup* at 30°C and 42°C. (E) Level changes of fatty acids upon temperature shift. (F) Comparison of temperature-induced level changes of fatty acid in *sup* and in the wild type or Δ*fabA*/*pTS-fabA*. i*, *P* < 0.05; *n* = 3.

To search for suppressors of Δ*fabA*, more than 10^9^ Δ*fabA*/*pTS-fabA* cells were spread out on LB plates and grown at the semirestrictive temperature of 40°C, permitting spontaneous mutations for 2 weeks (see Materials and Methods). A colony was found to contain only the deletion allele of *fabA* based on a PCR assay with sequence-specific primers (Fig. 5A). Hence, it was designated *sup* for suppressor of Δ*fabA*. A spot-plating assay and growth curve analysis confirmed that the *sup* strain rescued the growth defect of Δ*fabA* at a restrictive temperature (Fig. 5B and C). However, the *sup* mutant exhibited a slow-growth phenotype at 30°C.

Morphological analysis indicated that at a restrictive temperature, the *sup* mutant strain exhibited the curved cell form (Fig. 5D, see arrows) similar to that of Δ*fabA*/*pTS-fabA* (see Fig. 2). This result suggested that *sup* suppressed the growth defect but not the morphological defect of Δ*fabA* at a restrictive temperature. Notably, *sup* displayed a terminal phenotype of the filamentous form at a permissive temperature. These results implied that the *sup* mutant was a loss-of-function allele of an unknown gene required for growth: at a restrictive temperature, double mutation *sup^−^* Δ*fabA* survived due to suppression. At a permissive temperature, on the other hand, *sup* mutation exhibited slow growth with the filamentous phenotype. Alternatively, high-level upregulation of *sup* impeded wild-type cell growth, while mild-level upregulation rescued Δ*fabA* at a restrictive temperature.

FAME analysis indicated that only three major lipids were found in *sup*, namely, palmitoleic acid (C_16:1_), palmitic acid (C_16:0_), and stearic acid (C_18:0_). Unlike the wild type and Δ*fabA*/*pTS-fabA* (see Fig. 3A), the level of C_16:1_ at 42°C was not significantly decreased compared to that at 30°C (Fig. 5E, Table S2). The ratio of C_16:1_ between levels at 42°C and 30°C in *sup* was much less than that of Δ*fabA*/*pTS-fabA* (ratio in log_2_ scale: –0.55 in *sup* versus −2.51 in Δ*fabA*/*pTS-fabA*; *P* < 0.05, *n* = 3) (Fig. 5F). This result suggested that C_16:1_ retention in *sup* at a restrictive temperature was required for rescuing the growth defect of Δ*fabA*.

### Genome resequencing and transcriptomic profiling identified DesA as a candidate for overexpression suppressor of Δ*fabA* in *sup*.

**FIG 6:**
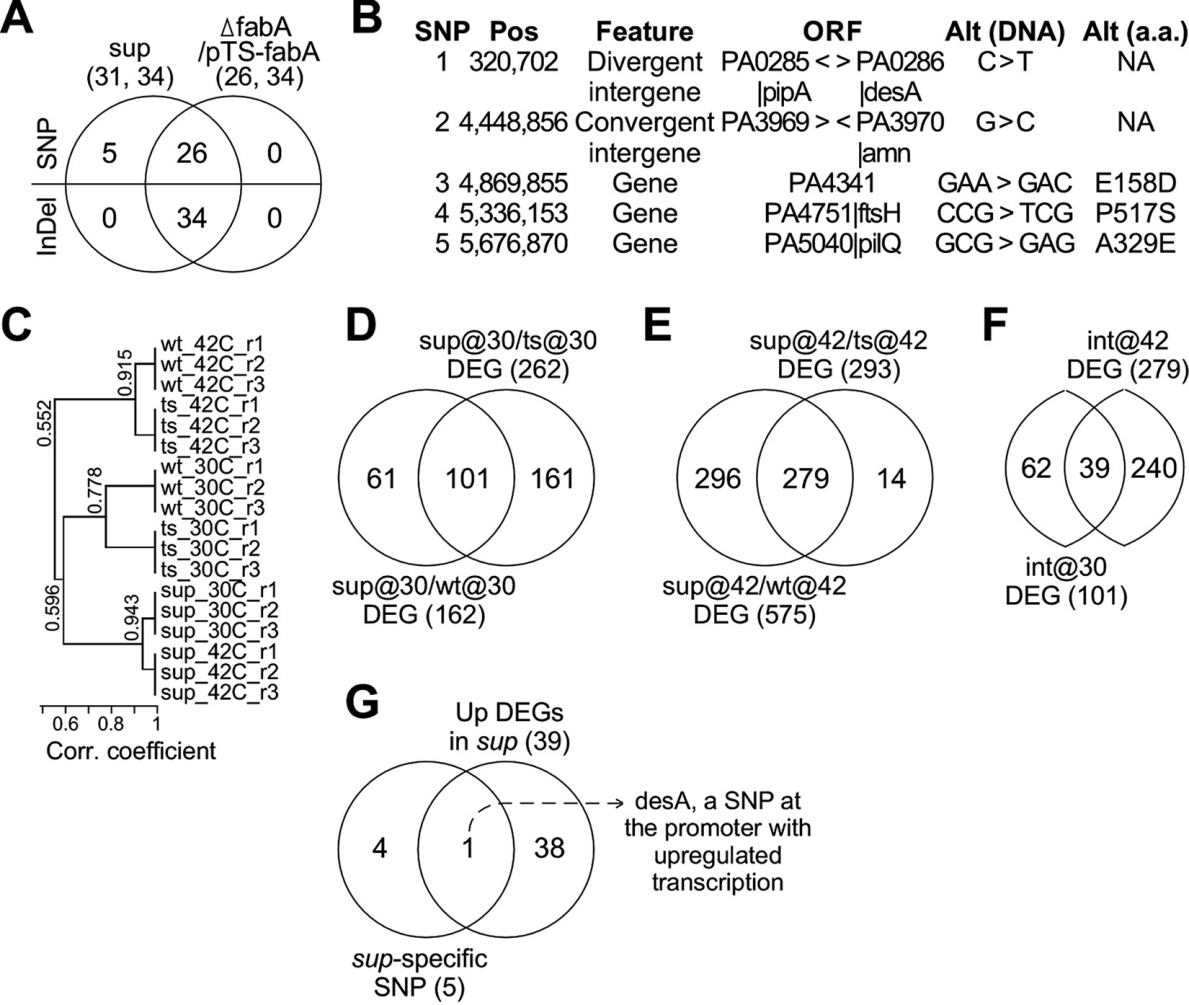
Identification of a candidate suppressor gene in *sup* by using genome resequencing and transcriptomic profiling. (A) Small mutations of SNP and indel loci detected using genome resequencing methodology; Venn diagram showing the mutation loci observed in *sup* and Δ*fabA*/*pTS-fabA*. (B) Locations of 5 supspecific SNP loci. SNP chromosome coordinates (Pos) and nucleotide alteration (Alt) are shown. (C) Correlations between transcriptional profiles of various samples. All samples are in triplicate. (D) Venn diagram showing the number of DEGs between *sup* versus Δ*fabA*/*pTS-fabA* and *sup* versus the wild type at 30°C. (E) Venn diagram showing the number of DEGs between *sup* versus Δ*fabA*/*pTS-fabA* and *sup* versus the wild type at 42°C. (F) Venn diagram showing the common genes between the genes in intersection at 30°C (B) and 42°C (C). (G) Venn diagram showing one (*desA*) of the 39 upregulated DEGs whose promoter contains a *sup*-specific SNP at the –64 nt position.

Genome resequencing was performed to identify candidate suppressor genes in the *sup* mutant compared to those in Δ*fabA*/*pTS-fabA* using Illumina technology (see Materials and Methods). Approximately 5 million 300-bp short reads were obtained for both *sup* and Δ*fabA*/*pTS-fabA*. The reads were mapped to the reference genome (http://www.pseudomonas.com) using Burrows-Wheeler Aligner (BWA) software (30) with an average depth of ~200× sequence coverage. A total of 65 small mutations (i.e., 31 SNP loci and 34 indel loci) were identified in *sup* using SAMtools (31), 60 (92.3%) of which already existed in Δ*fabA*/*pTS-fabA* (Fig. 6A, Table S3). This result suggested that 92.3% of the mutations in *sup* were not responsible for the suppression of Δ*fabA*. Of the five *sup* mutant-specific SNP loci, two were located at intergenes and three were in the coding sequences (Fig. 6B).

To investigate whether any of the *sup*-specific SNP-affecting genes would be significantly upregulated in *sup* that suppressed the lethality of Δ*fabA* at a restrictive temperature, we performed transcriptome sequencing (RNA-seq)-based transcriptomic profiling analysis in triplicate (Fig. 6C). We first identified subsets of 101 and 279 common differentially expressed genes (DEGs) (level increase, >2-fold; false-discovery rate (FDR)-adjusted *P* < 0.05, *n* = 3) in *sup* compared to that of the wild type and Δ*fabA*/*pTS-fabA* at 30°C and 42°C (Fig. 6D and E), respectively. A group of 39 DEGs were found in intersections of the two subsets of 101 and 279 DEGs (Fig. 6F, Table S4), indicating that these genes were upregulated in *sup* compared to the wild type and Δ*fabA*/*pTS-fabA* at 30°C and 42°C. Significantly, *desA*, one of the five *sup*-specific SNP-affecting genes was found in the subset of 39 upregulated DEGs in *sup* (*P* value = 0.038) (Fig. 6G). A SNP of C to T change located at the −64 nucleotide (nt) position of the *desA* promoter in *sup* could potentially be responsible for the alteration of *desA* transcription that fully suppressed the growth defect of Δ*fabA*. Similar to this observation, Zhu et al. (22) showed that a multicopy plasmid containing native promoter-controlled *desA* partially rescued the slow-growth phenotype of Δ*fabA*, in which *fabA* was not essential for viability (22). Hence, full suppression of the Δ*fabA* growth defect by *desA* overexpression needed to be experimentally validated.

### Δ*fabA* lethality is suppressed by an SNP-bearing promoter- or mild induced P_BAD_ promoter-controlled *desA*.

**FIG 7:**
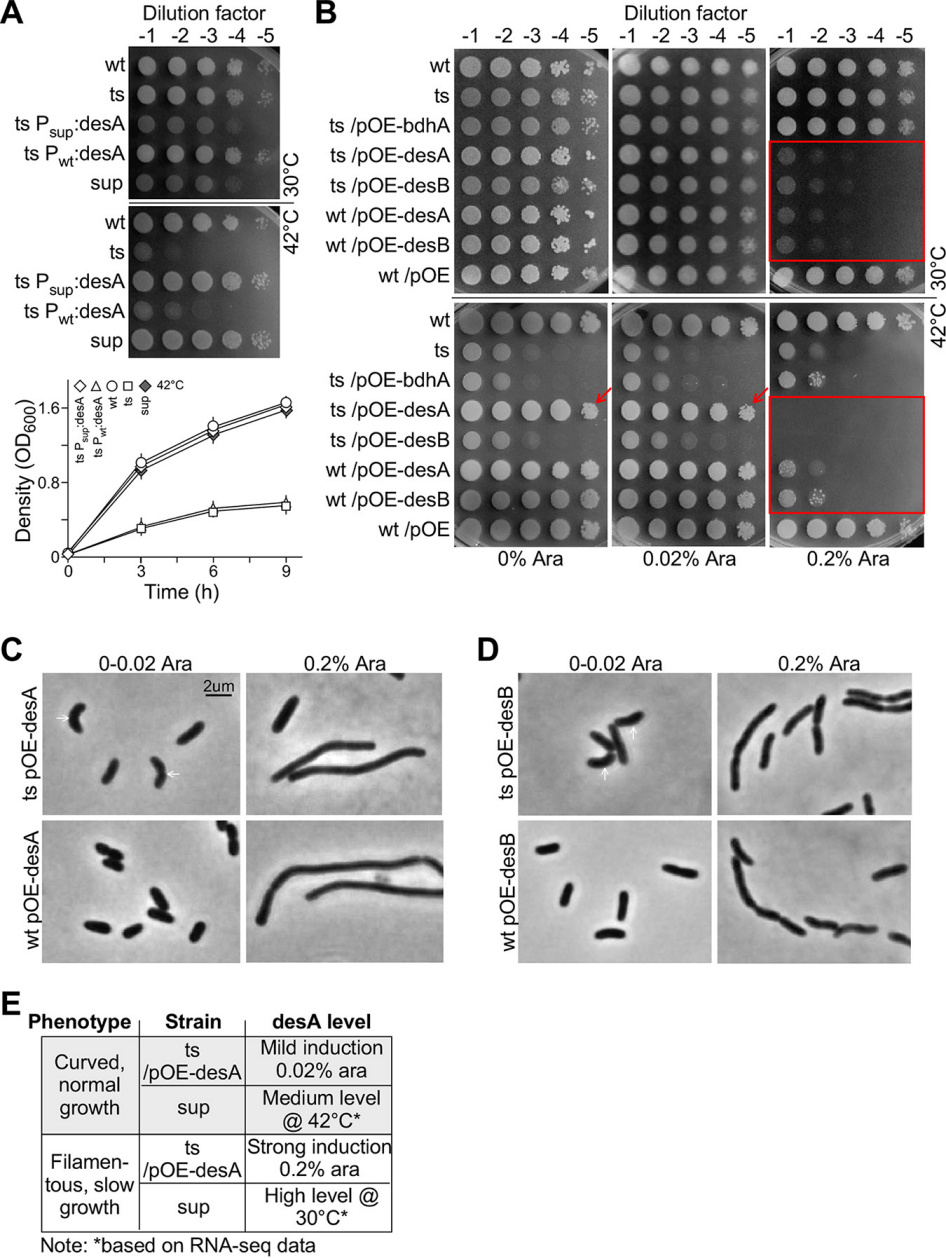
Mild induction of *desA-OE* but not *desB-OE* rescues the growth defect of Δ*fabA*/*pTS-fabA* at a restrictive temperature. (A) A point mutation at the promoter of *desA* is sufficient for Δ*fabA*/*pTS-fabA* to phenocopy the *sup* mutant. The top and bottom panels show the spot-plating assay and growth curve analysis, respectively. (B) Spot-plating assay. No induction (0 arabinose) or mild induction (0.02% arabinose) of *desA-OE* but not *bdhA-OE* or *desB-OE* rescues the growth defect of Δ*fabA*/*pTS-fabA* at a restrictive temperature (see arrows). Strong induction (0.2% arabinose) of *desA-OE* and *desB-OE* but not *bdhA-OE* hampers the growth of Δ*fabA*/*pTS-fabA* and wild-type cells (see rectangles). Wt/pOE served as the plasmid control. (C) Microscopic analysis of *desA-OE*. Images show the morphology of Δ*fabA*/*pTS-fabA* and wild-type cells at 42°C upon no or mild induction and strong induction of *desA-OE*. (D) Microscopic analysis of *desB-OE*. (E) Phenotype is dependent on the levels of *desA* in *sup* and Δ*fabA*/*pTS-fabA pOE-desA* strains. The relative *desA* transcription level in *sup* and Δ*fabA*/*pTS-fabA pOE-desA* is based on the RNA-seq data and arabinose induction, respectively.

Genome resequencing and transcriptomic profiling identified a SNP of C to T change at the −64 nt position in the promoter of *desA* whose transcription was upregulated in *sup* compared to that of Δ*fabA*/*pTS-fabA* and the wild type (see Fig. 6). To test if the point mutation at −64 nt in the promoter of *desA* was responsible for rescuing the growth defect of Δ*fabA*, we cloned the sequences of the promoter and coding region of *desA* from the *sup* mutant and wild type and integrated them into the genome of Δ*fabA*/*pTS-fabA* after validation by sequencing to generate strains of Δ*fabA P_sup_*:*desA*/*pTS-fabA* and Δ*fabA P_wt_*:*desA*/*pTS-fabA.* A spot-plating assay confirmed that a single point mutation at the promoter of *desA* was sufficient for Δ*fabA*/*pTS-fabA* to phenocopy the *sup* mutant. That is, Δ*fabA P_sup_*:*desA*/*pTS-fabA* but not Δ*fabA P_wt_*:*desA*/*pTS-fabA* exhibited slow growth at 30°C resembling that of the *sup* mutant (Fig. 7, top panel). On the other hand, growth of Δ*fabA P_sup_*:*desA*/*pTS-fabA* at 42°C was fully restored to that of the wild type and *sup*. Full growth restoration at 42°C was further validated using growth curve analysis (Fig. 7, bottom panel).

To investigate whether this was due to the alteration of *desA* transcription that rescued Δ*fabA*, we constructed the *desA-OE* plasmid pOE-*desA* and another desaturase *desB-OE* (22) plasmid, pOE-*desB*, in which target gene expression was under the control of the arabinose-regulated P_BAD_ promoter in the pBBR plasmid (See Materials and Methods). A plasmid, pOE-*bdhA*, for a gene that was involved in lipid metabolism (32) found in the group of 39 upregulated DEGs in the *sup* mutant was used as a control. These plasmids were transformed into the wild type and Δ*fabA*/*pTS-fabA* strains for growth analysis.

The spot-plating assay indicated that under no or mild induction (i.e., 0.02% arabinose supplementation), *desA-OE* but not *bdhA-OE* or *desB-OE* rescued the growth defect of Δ*fabA*/*pTS-fabA* at a restrictive temperature (Fig. 7B, see arrows). However, upon strong induction with 0.2% arabinose supplementation, *desA-OE* and *desB-OE* but not *bdhA-OE* repressed growth of Δ*fabA*/*pTS-fabA* and wild-type cells (Fig. 7B, see rectangles).

We subsequently examined the effect of *desA* and *desB* overexpression on the morphology of Δ*fabA*/*pTS-fabA* and wild-type cells. We found that under mild induction of 0.02% arabinose supplementation, *desA-OE* did not restore the morphology of Δ*fabA*/*pTS-fabA* at a restrictive temperature (Fig. 7C, see arrows). On the other hand, upon strong induction with 0.2% arabinose supplementation, both *desA-OE* and *desB-OE* induced a filamentous phenotype in Δ*fabA*/*pTS-fabA* and wild-type cells (Fig. 7C and D). However, cells with oleic acid supplementation did not exhibit filamentous morphology (see Fig. 3F), suggesting that only the increased level of DesA product unsaturated fatty acid (UFA)-containing phospholipid or DesB product UFA—coenzyme A (CoA) but not oleic acid altered the cell morphology. These results indicated that different levels of *desA* expression were attributed to the suppression phenotype of the *sup* mutant strain (Fig. 7E). They further implied that the levels of unsaturated fatty acid-containing membrane phospholipids played an essential role in the regulation of cellular morphology in P. aeruginosa.

### Δ*fabA* growth with supplementation of stearic acid is dependent on *desA* but not *desB*.

**FIG 8:**
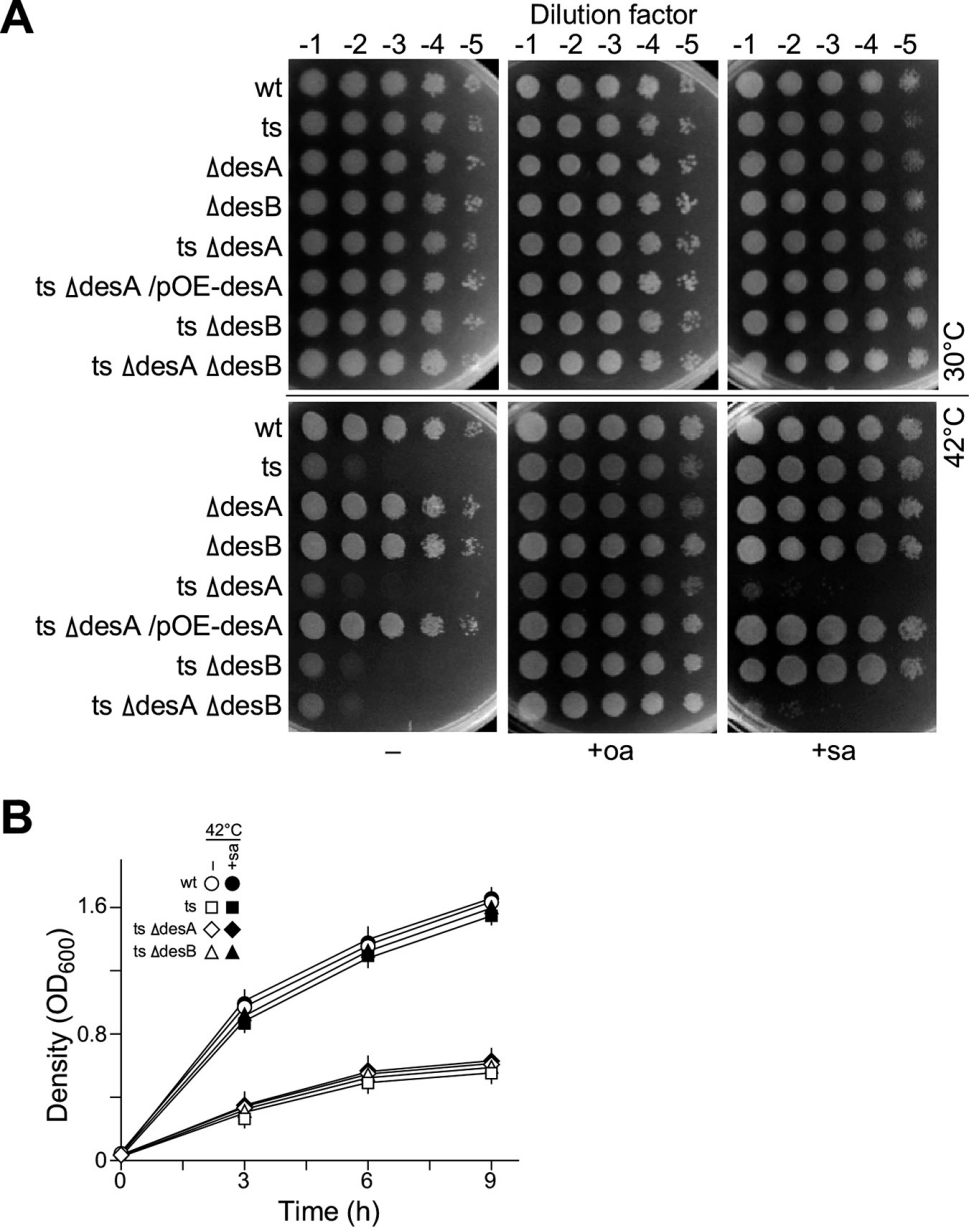
Δ*fabA*/*pTS-fabA* auxotrophic for stearic acid is dependent on *desA* function but not *desB*. (A) The spot-plating assay shows various strains growing on a plate with oleic acid (oa) or stearic acid (sa) supplementation. (B) Growth curve analysis of various strains indicated at 42°C in medium with or without stearic acid.

DesA and DesB were found to be dispensable desaturases in P. aeruginosa (22). We wanted to test if Δ*fabA* auxotrophic for oleic acid (i.e., UFA) and stearic acid (i.e., saturated fatty acid [SFA]) required the function of *desA* or *desB*. For this reason, we constructed single mutants Δ*desA* and Δ*desB*, double mutants Δ*fabA* Δ*desA*/*pTS-fabA* and Δ*fabA* Δ*desB*/*pTS-fabA*, and triple mutant Δ*fabA* Δ*desA* Δ*desB*/*pTS-fabA* (see Materials and Methods, Fig. S3). These strains were subjected to a spot-plating assay on LB plates supplemented with oleic acid, stearic acid, or no fatty acid as control at 30°C and 42°C. The results indicated that Δ*fabA*/*pTS-fabA* and Δ*fabA* Δ*desB*/*pTS-fabA* grew on LB plates supplemented with oleic acid or stearic acid at 42°C (Fig. 8A). On the other hand, Δ*fabA* Δ*desA*/*pTS-fabA* and Δ*fabA* Δ*desA* Δ*desB*/*pTS-fabA* grew on plates supplemented with oleic acid but not stearic acid at 42°C. The growth at 42°C was also tested using growth curves (Fig. 8B). These results indicated that Δ*fabA* auxotrophic for stearic acid was dependent on the function of *desA* but not *desB*. This was supported by the observation that mild induction of *desA-OE* but not *desB-OE* rescued the growth defect of Δ*fabA* (see Fig. 7B). In contrast, Zhu et al. (22) showed that Δ*fabA* Δ*desA* but not Δ*fabA* Δ*desA* Δ*desB* grew in medium supplemented with stearic acid, concluding that Δ*fabA* Δ*desA* auxotrophic for SFA was *desB* dependent, increasing the level of discrepancy between the two studies. It would be interesting discover the factors in the background that led to the discrepancies in these analyses.

## DISCUSSION

Fatty acids are essential for bacterial cell viability (10). That is why microbial enzymes involved in type II fatty acid synthesis (FASII) are popular targets for antibacterial drug development (11, 12). Pseudomonas aeruginosa is an opportunistic pathogen for which new drug development is needed (1–3). *ts*-Plasmid-based conditional alleles of essential genes are useful for deletion analysis of essential genes of interest in P. aeruginosa (8). However, multicopy *ts*-plasmids hamper the analysis of the mutant phenotype because cells deplete the *ts*-plasmid at the onset of the stationary phase after the shift to a restrictive temperature. Mutant cells at the stationary phase slow down the phenotype development. In this study, we developed a consecutive subculture protocol to ensure the rapid growth of mutant cells prior to the point of plasmid depletion (see Fig. S1).

Many essential genes are suppressible (6, 9). Hence, it is possible to accumulate spontaneous suppressors during construction of essential gene deletion strains without protection of complementary copies. It is also possible that different strain backgrounds can lead to the difference of *fabA* essentiality in P. aeruginosa (21, 22). To avoid suppressor accumulation, we deleted the *fabA* chromosomal copy in the presence of a complementary copy in the *ts*-plasmid. The resulting Δ*fabA*/*pTS-fabA* strain failed to grow at a restrictive temperature under aerobic conditions (see Fig. 1).

FabA is bifunctional enzymes 3-hydroxyacyl-ACP dehydratase and *trans*-2-enoyl-ACP isomerase, which are involved in the synthesis of saturated and unsaturated fatty acids, respectively (13–16). FabZ, another copy of 3-hydroxyacyl-ACP dehydratase, shares functions in cycles of fatty acid elongation with FabA involved in saturated fatty acid synthesis (16). In this study, we show that mild induction of *fabZ-OE* does not rescue the Δ*fabA*/*pTS-fabA* growth defect at a restrictive temperature, suggesting that the growth defect of Δ*fabA*/*pTS-fabA* is fully attributed to the loss of unsaturated fatty acid synthesis (see Fig. 4).

Essential genes can be bypass-suppressed (6, 9). In this case, transcription repression-based analysis of essential genes is unsuitable for suppressor analysis, because the repression machinery tends to accumulate mutations upon extended growth (33, 34). By using the *ts*-plasmid-based Δ*gmhB*/*pTS-gmhB* strain, we previously identified the *fbp* gene, whose overexpression suppresses the growth defect of Δ*gmhB*/*pTS-gmhB* at a restrictive temperature (8). In this study, by using the same approach, we identified a point mutation at the promoter of the desaturase DesA in *sup*, which is sufficient for Δ*fabA*/*pTS-fabA* to phenocopy the *sup* mutant that exhibits slow growth at 30°C and normal growth at 42°C (see Fig. 7A), consistent with growth phenotypes by strong overexpression and mild overexpression of *desA*, respectively (see Fig. 7B). Similar to this, Zhu et al. proposed that oleic acid repressed the *desA* expression in Δ*fabA* and showed that multicopy *desA* partially alleviated the slow growth phenotype of Δ*fabA* in medium without oleic acid supplementation (22). It was not clear whether the discrepancy of *fabA* essentiality could be caused by a spontaneous suppressor prior to or during strain construction. We propose that the plasmid-based *ts*-alleles of essential genes are useful for systematic deletion and suppressor analyses of essential genes of interest in P. aeruginosa.

Unsaturated fatty acid is believed to modulate membrane fluidity; e.g., at high (i.e., 42°C) and low (i.e., 30°C) temperatures, levels of unsaturated fatty acid in membrane lipids decrease and increase, respectively (35). In this study, we show that cells display curved morphology upon depletion of FabA, suggesting that disruption of membrane fluidity homeostasis leads to the loss of polarized growth (see Fig. 2 and 3). Furthermore, cells exhibit oval morphology after strong overexpression of *fabA* and *fabZ* (see Fig. 4). Given the identical effects of *fabA-OE* and *fabZ-OE*, the oval cell morphology phenotype is unlikely to be related to the synthesis of unsaturated fatty acid. We propose that a high level of FabA or FabZ activity depletes its substrate 3-hydroxyacyl-ACP, which in turn limits the synthesis of lipid A, a major cell wall lipopolysaccharide component in Gram-negative bacteria (36). Disruption of *fabZ* is known to suppress the growth defect of *lpxA^−^* and *lpxC^−^* mutants that are incapable of synthesizing lipid A (37).

Although mild induction of *desA-OE* suppresses the growth defect of Δ*fabA*/*pTS-fabA* at a restrictive temperature, strong overexpression of *desA* impedes cell growth with a filamentous phenotype (see Fig. 7). It is known that *desA* that utilizes phospholipid but not free fatty acid as a substrate to increase the level of unsaturated double bonds in fatty acyls (22). Hence, this result implies that when the level of unsaturated fatty acid in membrane lipid is higher than usual, rod-shaped cells will turn into filamentous forms.

While DesA is an *sn*-2-position phospholipid Δ9-desaturase, DesB is a proposed acyl-CoA Δ9-desaturase that permits the growth of Δ*fabA* Δ*desA* in medium supplemented with saturated fatty acid (22). In this study, however, we show that Δ*fabA* Δ*desA/pTS-fabA* does not grow on medium with stearic acid supplementation regardless of the presence of *desB* (see Fig. 8), which is supported by the observation that *desB-OE* does not rescue the Δ*fabA*/*pTS-fabA* lethality at a restrictive temperature (see Fig. 7). These results increased the level of discrepancies between the two studies (22; this study). Spontaneous mutation without an apparent phenotype often went unnoticed (23). However, when a mutation with a growth advantage occurs, it will be quickly enriched in the population, such as in an essential gene-deletion mutant during construction. Hence, the preexistence or enrichment of a spontaneous mutation or suppressor is a complex issue that warrants further study.

To summarize the similarities and differences between this study and the previously reported studies (21, 22), we show that when using the *ts*-plasmid-based conditional mutant strain Δ*fabA*/*pTS-fabA*, *fabA* is essential for growth under aerobic conditions in P. aeruginosa, similar to the study by Hoang and Schweizer (21) but different from that by Zhu et al. (22). Δ*fabA*/*pTS-fabA* cells exhibit curve morphology at a restrictive temperature. Mild overexpression of *fabZ*, a *fabA* isozyme, does not rescue the growth defect of Δ*fabA*. However, strong overexpression of *fabA* or *fabZ* impedes growth of cells displaying oval morphology. From the spontaneous mutagenesis screening, we isolated a suppressor, *sup*, that fully rescues the growth defect but not the curved morphology of Δ*fabA* at 42°C. On the other hand, *sup* exhibits a slow-growth phenotype with filamentous morphology at 30°C. Genome resequencing and transcriptomic profiling of *sup* compared to that of Δ*fabA*/*pTS-fabA* identifies *desA*, whose promoter bears a SNP at the −64 position, and the transcription level is significantly upregulated compared to that of Δ*fabA*/*pTS-fabA* and the wild type. We validate that integration of the SNP-bearing promoter-controlled *desA* is sufficient for Δ*fabA*/*pTS-fabA* to phenocopy the *sup* mutant. Furthermore, mild overexpression of *desA* fully rescues the growth defect but not morphology of Δ*fabA.* It is interesting to note that strong overexpression of *desA* impedes cell growth with a filamentous morphology, resembling the *sup* phenotype at 30°C. Zhu et al. (22) characterized the two desaturases, *desA* and *desB*, in P. aeruginosa. They propose that *desA* expression is repressed in the presence of oleic acid and show that multicopy *desA* can partially rescue the slow-growth phenotype of Δ*fabA* without oleic acid (22). They further propose that Δ*fabA* Δ*desA* auxotrophic for SFA such as stearic acid and palmitic acid is *desB* dependent (22). In this analysis, we show that Δ*fabA/pTS-fabA* and Δ*fabA* Δ*desB/pTS-fabA* grow in medium with stearic acid supplementation at 42°C. In contrast, Δ*fabA* Δ*desA/pTS-fabA* and Δ*fabA* Δ*desA* Δ*desB*/*pTS-fabA* do not grow, indicating that Δ*fabA* auxotrophic for SFA is *desA* dependent. Consistent with this, we show that mild expression of *desB* does not rescue the growth defect of Δ*fabA*. These observed discrepancies could be caused by the differences between the strain backgrounds (23). It would be interesting in the future to identify the background factor that leads to the differences between the two strains.

## MATERIALS AND METHODS

### Oligonucleotides, plasmids, and bacterial strains.

The oligonucleotides, plasmids, and bacterial strains used in this study are listed in Table 1. The P. aeruginosa PAO1 wild-type strain (BioSciBio, Hangzhou, China) and its derivatives were cultivated in LB (in 1 L: 10 g tryptone, 10 g NaCl, 5 g yeast extract, pH 7.0) liquid or solid (addition of 1.5% agar) medium supplemented with antibiotics (e.g., 100 μg mL^−1^ ampicillin, 50 μg mL^−1^ gentamicin, and 100 μg mL^−1^ tetracycline) and chemicals (e.g., 15% sucrose or 0.002%, 0.02%, or 0.2% arabinose) at 30°C or 42°C as indicated. Temperature-sensitive mutant strains were maintained in LB medium with adequate supplementation at the permissive temperature of 30°C. The chemicals used in this study were purchased from Sigma-Aldrich Co. (St. Louis, MO, USA).

**TABLE 1:** DNA, plasmids, and strains used in this study

Name	Seq (5′–3′)	Comment
Oligonucleotides		
fabA_up_F	tcgaagcttgcatgcctgcagGCGTGGCCAGGCAGGCCGATA	FabA upstream fragment
fabA_up_R	cggtaactagtcagtcataagccAGTGCCGAAGGCCTCCGCGT	
fabA_dn_F	ggcttatgactgactagttaccgGCGACTGCAGCGCAGCAGGT	FabA downstream fragment
fabA_dn_R	ggcaaatattatacgggtaccCTGCCGCCTGCCGGTGCTCGA	
fabA_comp_F	cttgcggagaactgtggtaccCTTGCCCAGCTGGATTTGTTC	FabA expression sequence
fabA_comp_R	tagatgcattcgcgaggtaccATCCGGTGAACCAGACAGTCATAG	
fabA_F1	GGAAACGGTGTCTTTGTCATTGC	Assay fabA chromosome/plasmid allele
fabA_R1	CCGCAACGCAACAGTCTATGAC	
fabA_F2	GACGATCTCGGCGTAGATCTTG	Assay *fabA* chromosome allele
fabA_R2	ACCCTGCAGGCAGAGAGCAA	
Plasmids		
DEL-plasmid	pUC19-Gm^R^-sacB	8
RES-plasmid	pUC57-Tc^R^-rep^ts^	8
OE-plasmid	pBBR1MCS5-araC-P_BAD_ or pBBR-P_BAD_	This study
*fabA* DEL-plasmid	pDEL-Δ*fabA*	This study
*fabA* RES-plasmid	pRES-*fabA*	This study
*fabA*-OE plasmid	pBBR-P_BAD_:*fabA*	This study
*bdhA*-OE plasmid	pBBR-P_BAD_:*bdhA*^+^	This study
*fabZ*-OE plasmid	pBBR-P_BAD_:*fabZ*^+^	This study
*desA*-OE plasmid	pBBR-P_BAD_:*desA*^+^	This study
Strains		
DH5α	E. coli DH5α	8
PAO1	P. aeruginosa PAO1, wild type	8
Δ*fabA*/*pTS-fabA*	Δ*fabA*/*pRES-fabA*	This study
Δ*fabA*	Δ*fabA* in oleate medium	This study
Δ*fabA*/*pTS-fabA pOE-fabA*		This study
Δ*fabA*/*pTS-fabA pOE-fabZ*		This study
Δ*fabA*/*pTS-fabA pOE-desA*		This study
Δ*fabA*/*pTS-fabA pOE-desB*		This study
Δ*fabA*/*pTS-fabA pOE-bdhA*		This study
*sup*	Δ*fabA sup*	This study
Δ*fabA* Δ*desA*/*pTS-fabA*		This study
Δ*fabA* Δ*desB*/*pTS-fabA*		This study
Δ*fabA* Δ*desA* Δ*desB*/*pTS-fabA*		This study
Δ*fabA* P_sup_:desA*/pTS-fabA*		This study
Δ*fabA* P_wt_:desA*/pTS-fabA*		This study

### Growth curve of consecutive subcultures for assessment of the plasmid-based *ts*-mutant.

The complementary plasmid is a multicopy plasmid in the Δ*fabA*/*pTS-fabA* strain (38). We found that 4.5 h after the shift to the restrictive temperature, cells start to show average copy numbers of plasmid close to those of the chromosome or *ts*-plasmid nearing depletion (Fig. S1). However, slow-growing stationary-phase cells hamper assessment of the mutant phenotype. To circumvent this issue, we adopted the consecutive subculture method (39) for the analysis of the Δ*fabA*/*pTS-fabA* growth curve (39). In brief, the fresh overnight culture at 30°C was inoculated into a shake flask to a starting OD_600_ of 0.05 at 42°C (see Fig. S1). Cell growth was monitored by measuring the OD at various time points. Growth of Δ*fabA*/*pTS-fabA* slowed down after several generations at 4.5 h at 42°C compared to that of the wild type. We took the first subculture at 4 h the as inoculum to start the second subculture for the analysis of the Δ*fabA*/*pTS-fabA* growth curve. Based on the ratio of plasmid and chromosome copy numbers, it was clear that depletion occurred after starting the second subculture. Hence, the growth curve of the second subculture was used for the Δ*fabA*/*pTS-fabA* mutant strain.

### Plasmid construction.

Deletion and rescue plasmids (8) were used for construction of the pDEL-Δ*fabA* and pRES-*fabA* plasmids, respectively (see Fig. 1A). Briefly, for pDEL-Δ*fabA* plasmid construction, a Δ*fabA* deletion cassette consisting of 500 bp upstream and 500 bp downstream sequences was PCR amplified using oligonucleotides expanded with overlapping sequences for cloning using a cloning kit (ClonExpress II one-step cloning kit, Vazyme, China). After double digestion with PstI and KpnI, pDEL and the *fabA* upstream and downstream sequences were subjected to cloning according to the manufacturer’s instruction (Vazyme). For pRES-*fabA* plasmid construction, the native promoter containing the *fabA* coding sequence was PCR amplified with oligonucleotides expanded with overlapping sequences with the vector cloning site. After KpnI digestion, the pRES plasmid and the native promoter-controlled *fabA* sequence were subjected to Vazyme cloning. For gene overexpression plasmids, pBBR1MCS-5 (or pBBR) was utilized (40). Briefly, the *araC*-P_BAD_ promoter sequence (29) and the target gene sequence were PCR amplified using oligonucleotides expanded with overlapping sequences for Vazyme cloning. The BamHI and PstI double-digested pBBR plasmid, together with *araC*-P_BAD_ and the target gene sequences, was subjected to Vazyme cloning. All constructed plasmids were sequencing-validated prior to use.

### Strain construction.

We adopted the three-step protocol (8) to construct the plasmid-based *ts*-allele of the Δ*fabA*/*pTS-fabA* strain. Briefly, we first electroporated the pDEL-Δ*fabA* plasmid into the P. aeruginosa PAO1 strain and isolated the plasmid integrants via a single crossover into the genome on a gentamicin (Gm)-containing LB plate, because the pDEL plasmid could not be autoreplicated in P. aeruginosa. Second, a pRES-*fabA* plasmid was transformed into the pDEL-Δ*fabA* plasmid integrant on the tetracycline-containing LB plate. Third, the resulting transformants were subjected to counterselection of *sacB* for generation of the chromosomal Δ*fabA* allele after looping out the integrated pDEL-Δ*fabA* plasmid via single crossover on a sucrose-containing LB plate. The plasmid-based Δ*fabA*/*pTS-fabA* strains were PCR validated for the chromosomal Δ*fabA* allele and spot-plating assay for the *ts*-growth phenotype. For deletion of nonessential genes such as Δ*desA* and Δ*desB*, only the deletion plasmid was applied. For construction of a strain containing a chromosomal copy of *desA-C-64T* (nucleotide C to T change at the −64 nt position of *desA* as in the *sup* mutant), *desA* promoter and coding sequences were PCR amplified from *sup* and wild-type cells (as control) and cloned into an integration vector such as the deletion plasmid. The resulting plasmid was transformed into the Δ*fabA*/*pTS-fabA* strain to yield Δ*fabA P_sup_*:*desA*/*pTS-fabA* and Δ*fabA P_wt_*:*desA*/*pTS-fabA.*

### DNA transformation.

For P. aeruginosa strain construction, electrocompetent cells were prepared using the protocol of Huang and Wilks (41) with minor modifications. Briefly, 5 mL log-phase cells was harvested and washed with 10% glycerol three times. Subsequently, the cells were resuspended in 10% glycerol. For electroporation, 90 μL of electrocompetent cells mixed with 10 μg of plasmid DNA was transferred to an electroporation cuvette with a 1-mm gap (Bio-Rad) and a pulse of 1,200 V, 2.5 mF, and 5 ms was applied using a Bio-Rad Xcell electroporator. After the pulse, 1 mL of LB medium was immediately added to the cuvette and mixed gently, and the mixture was transferred to a fresh tube and incubated at 30°C for 3 h with shaking at 200 rpm before plating onto LB plates supplemented with the appropriate antibiotics. Transformants usually appeared after overnight incubation.

### Spot-plating assay.

The spot-plating assay (42) was adopted to test sensitivities to stress factors such as antibiotics, sucrose, and temperature. Briefly, 10-fold serial-diluted cultures were transferred using a 48-pin replicator (V&P Scientific, Inc.) onto LB plates supplemented with appropriate stress factors and incubated at 30 or 42°C as indicated.

### Fluorescence microscopy.

Cell morphology was investigated under a BX53 microscope (Olympus, Tokyo, Japan) using the phase contrast configuration. For the cell outline cytoplasmic membrane, Nile red staining was employed. Briefly, 50 μL overnight culture was added to 5 mL LB broth and grown in a shaker to an OD_600_ of 0.8 for examination at 30°C and at 6 h and 9 h after the shift to 42°C. From the resulting fresh culture, 1 mL was harvested and resuspended with 4% formaldehyde fixative solution. After the cells were fixed in the formaldehyde solution for 30 min or more at room temperature, the fixed cells were washed with phosphate-buffered saline (PBS) (in 1 L: 10.9 g Na_2_HPO_4_, 3.2 g NaH_2_PO_4_, and 90 g NaCl, Ph7.4) and were ready for fluorescence dye staining. Cell suspension was added with Nile red at a final concentration of 10 ng mL^−1^ for 30 min and then washed and resuspended in PBS for fluorescence microscopic examination.

### Lipid extraction and fatty acid methyl ester (FAME) preparation.

Cellular lipids were extracted using a chloroform-methanol solution (2:1 vol/vol). The organic phase was transferred to a fresh tube and blown with nitrogen gas to evaporate organic solvent. The resulting lipid was weighted as the quantity of total lipids and resuspended in hexane to a desired concentration. For analysis of fatty acid composition, one part of the extracted lipids was transesterified with methanol to generate fatty acid methyl ester (FAME) according to a published protocol (27). The total lipid extracted from P. aeruginosa using chloroform methanol (2:1 vol/vol) solution is known to contain neutral and phospholipids that predominantly possess hexadecanoic (C_16:0_), hexadecenoic (C_16:1_), octadecanoic (C_18:0_), octadecenoic (C_18:1_), 17- and 19-cyclopropane, etc. acids (25, 26).

### Gas chromatography coupled with mass spectrometry (GC-MS) analyses.

To determine the FAME species, 1 μL FAMEs was directly injected into the injection port of a gas chromatograph (2010Plus GC system, Shimadzu Co., Tokyo, Japan) coupled with a mass spectrometer (MS) system (Shimadzu QP2020 with quadrupole analyzer). The GC was operated on an Rtx-5MS GC column (30 m × 0.25 mm inside diameter [i.d.] with 0.25-μm film thickness of 5%-phenyl-methylpolysiloxane) (Restek Co., Bellefonte, PA, USA), and helium (purity, 99.999%) was used as the carrier gas. The temperature of the injection port was set to 260°C, while the sample injection was made in splitless mode with a purge flow of 50 mL min^−1^ for 1 min. The temperature program was started with an initial temperature of 160°C and then was increased 2°C min^−1^ to 230°C for 10 min. The mass spectrometer was operated in electron ionization (EI) mode with the ion source temperature set at 230°C. The electron energy was 70 eV. Full-scan MS data were acquired in the range of 50 to 500 *m/z* to obtain the fragmentation spectra of the FAMEs. LabSolutions (Shimadzu Co.) was used to determine all the peaks in the raw GC chromatogram. A library search was done for all the peaks using the National Institute of Standards and Technology NIST/EPA/NIH (NIST 14 Library).

### Isolation of suppressors.

Approximately 10^9^ Δ*fabA*/*pTS-fabA* cells were plated onto LB plates and incubated at the semirestrictive temperature of 40°C for 2 weeks for suppressors through spontaneous mutations as described (8, 9). During the incubation, plates were placed in a bag with a stream of fresh air filtered with a 0.22-μm-pore-size filter after passing through a water container to maintain the humidity and oxygen. Suppressor colonies were validated via streaking on fresh LB plates and incubated at 42°C. Primer-specific PCR assays for the deletion allele were also performed for validation of the *fabA* deletion allele.

### Genome resequencing analysis.

Genomic DNA was extracted with a Genomic DNA extraction kit (TaKaRa Bio, Inc.) and sheared to ~400 bp in length using an S2 instrument (Covaris, Woburn, MA, USA). The sequencing library was constructed using s NEXTflex DNA sequencing kit (Bioo Scientific, USA) according to the package instructions and then was sequenced on a MiSeq PE300 device (Illumina, Inc., San Diego, CA, USA), generating a total of 5 million clean reads of 300 bp in length for both *sup* and Δ*fabA*/*pTS-fabA* (BioMedical Institute of Shanghai, Shanghai, China). The reads were mapped to the reference genome PAO1 (http://www.pseudomonas.com) using BWA software (30). Analysis of small sequence variants such as single-nucleotide polymorphisms (SNPs) and small insertions and deletions (indels) of less than 50 bp was performed using the SAMtools software (31).

### RNA-seq-based transcriptomic analysis.

Total RNA was extracted from various cell samples in triplicate with an RNA extraction kit (TaKaRa Bio, Inc.). After a qualification check with a 2100 Bioanalyzer (Agilent Technologies, Inc., Redwood City, CA, USA), qualified samples were treated with 10 U DNase I (TaKaRa) at 37°C for 30 min. The resulting RNA was subjected to rRNA removal using a Ribo-Zero magnetic kit (for Gram-negative bacteria) (Epicentre Biotechnologies, Inc., Madison, WI, USA) following the manufacturer’s instructions. rRNA-depleted RNA was used for RNA-seq analysis using the Illumina HiSeq 2500 at the Shanghai Human Genome Centre (Shanghai, China). Briefly, 100 ng rRNA-depleted RNA was used for construction of sequencing libraries using the NEBNext Ultra directional RNA library prep kit according to the manufacturer’s instructions. The acquired raw data were processed using fastp software (43) to generate the clean reads that were mapped to the reference genome (http://www.pseudomonas.com) using Salmon (39). The transcription level was normalized to transcripts per kilobase per million mapped reads (TPM). Differentially expressed genes (DEGs) were defined based on a level of change of >2-fold and a *P* value of <0.05 using DESeq2 (44).

### Statistical analysis.

A binomial test was applied to determine the nonrandom distribution. *P* values derived from multiple testing were adjusted using the Benjamini-Hochberg method (45). Differences between the means of two and more subgroups were tested using *t* test and one-way analysis of variance (ANOVA), respectively.

### Data availability.

The RNA-seq raw data sets were submitted to the NCBI database with the accession number PRJNA917454.
