# Supplementary material for: Analysis of the Plasmid-Based ts-Mutant ΔfabA/pTS-fabA Reveals Its Lethality under Aerobic Growth Conditions That Is Suppressed by Mild Overexpression of desA at a Restrictive Temperature in Pseudomonas aeruginosa
Source: Microbiol Spectr. 2023 May 16;11(3):e01338-23. doi: 10.1128/spectrum.01338-23 (PMC10269440; doi:10.1128/spectrum.01338-23)
Supplement: Supplemental file 1 — Supplemental material. Download spectrum.01338-23-s0001.pdf, PDF file, 1.8 MB [file spectrum.01338-23-s0001.pdf]

Supplementary Figure S1. Growth curve of the subcultures. (A) Schematic successive subcultures. (B) Growth curve of the first subculture with a starting OD of 0.05. (C) Growth curve of the second subculture with a starting OD of 0.05. (D) Cell morphology at various time points in subcultures. (E) Ratio of copy numbers between ts-plasmid and chromosome in various cell samples based on RT-PCR assay using plasmid and chromosome sequence-specific primers.

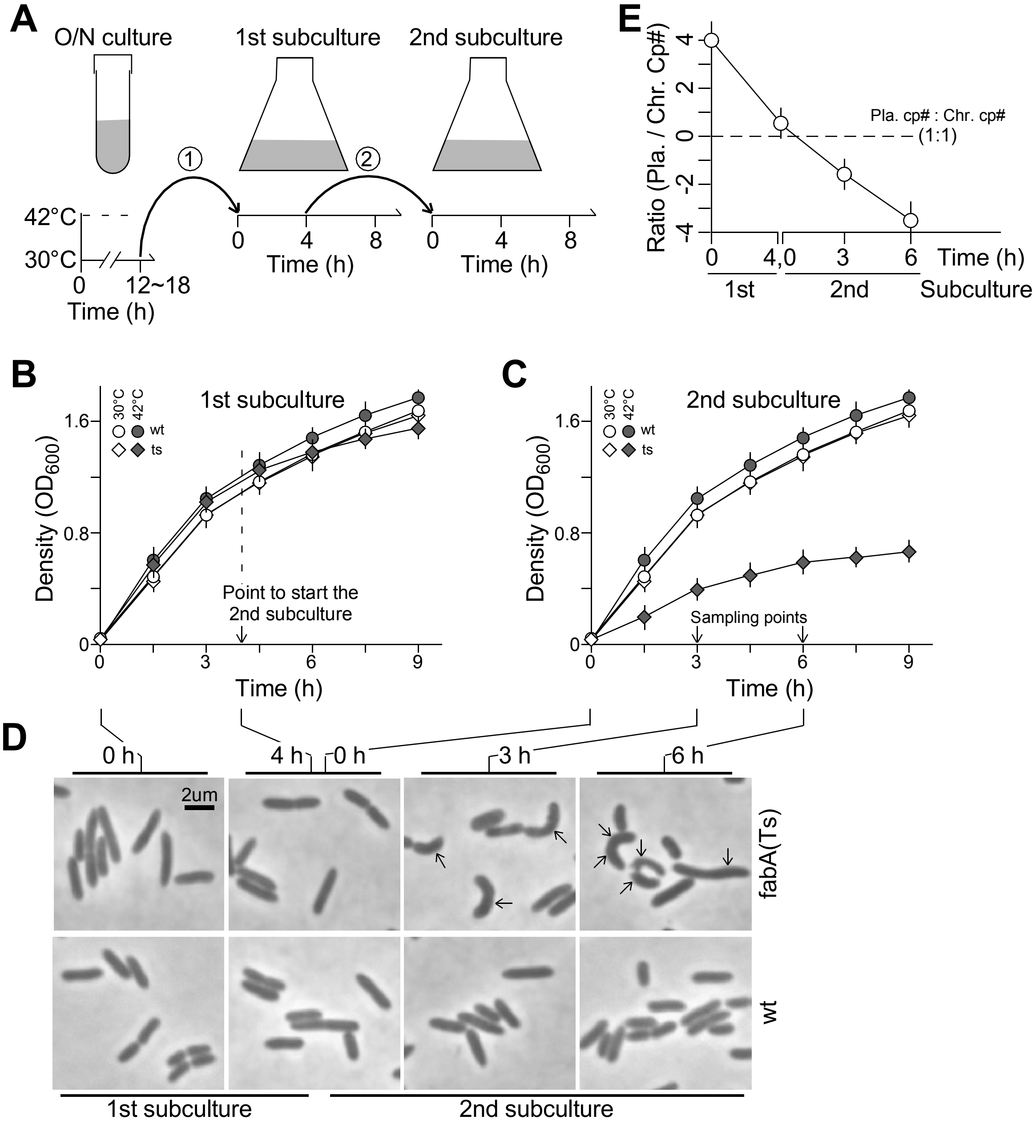

Supplementary Figure S2. GC-MS analysis of FAME in fabA(Ts) and wild type at 30°C and 42°C. (A) GC chromatograms. (B) MS spectra.

A

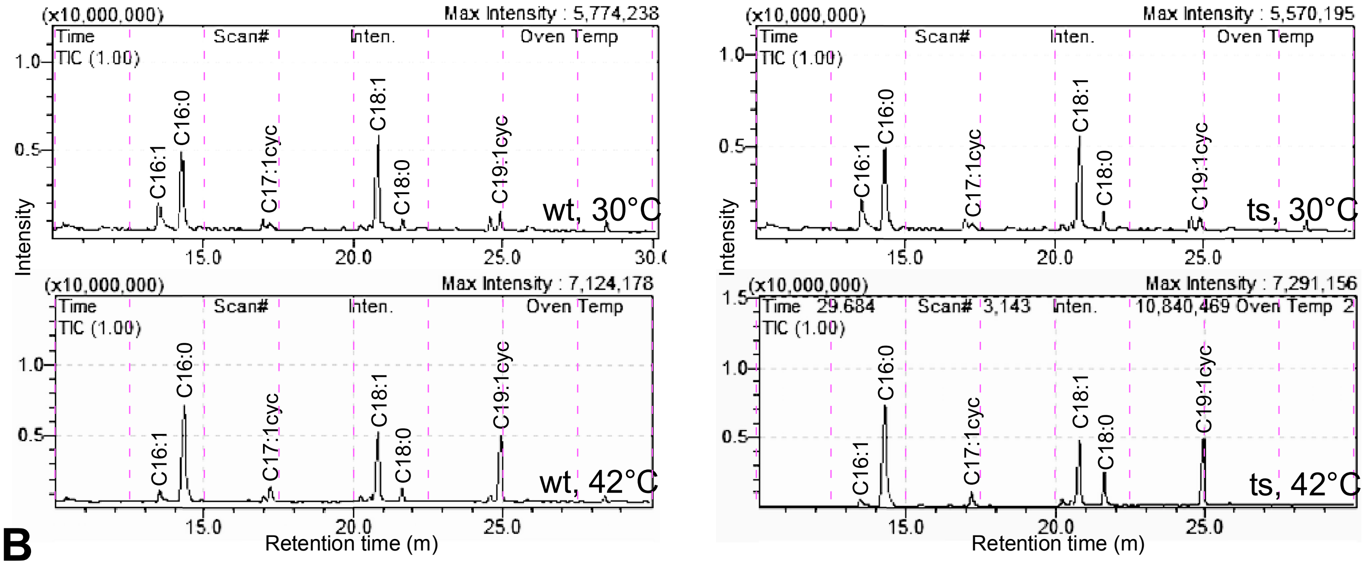

B

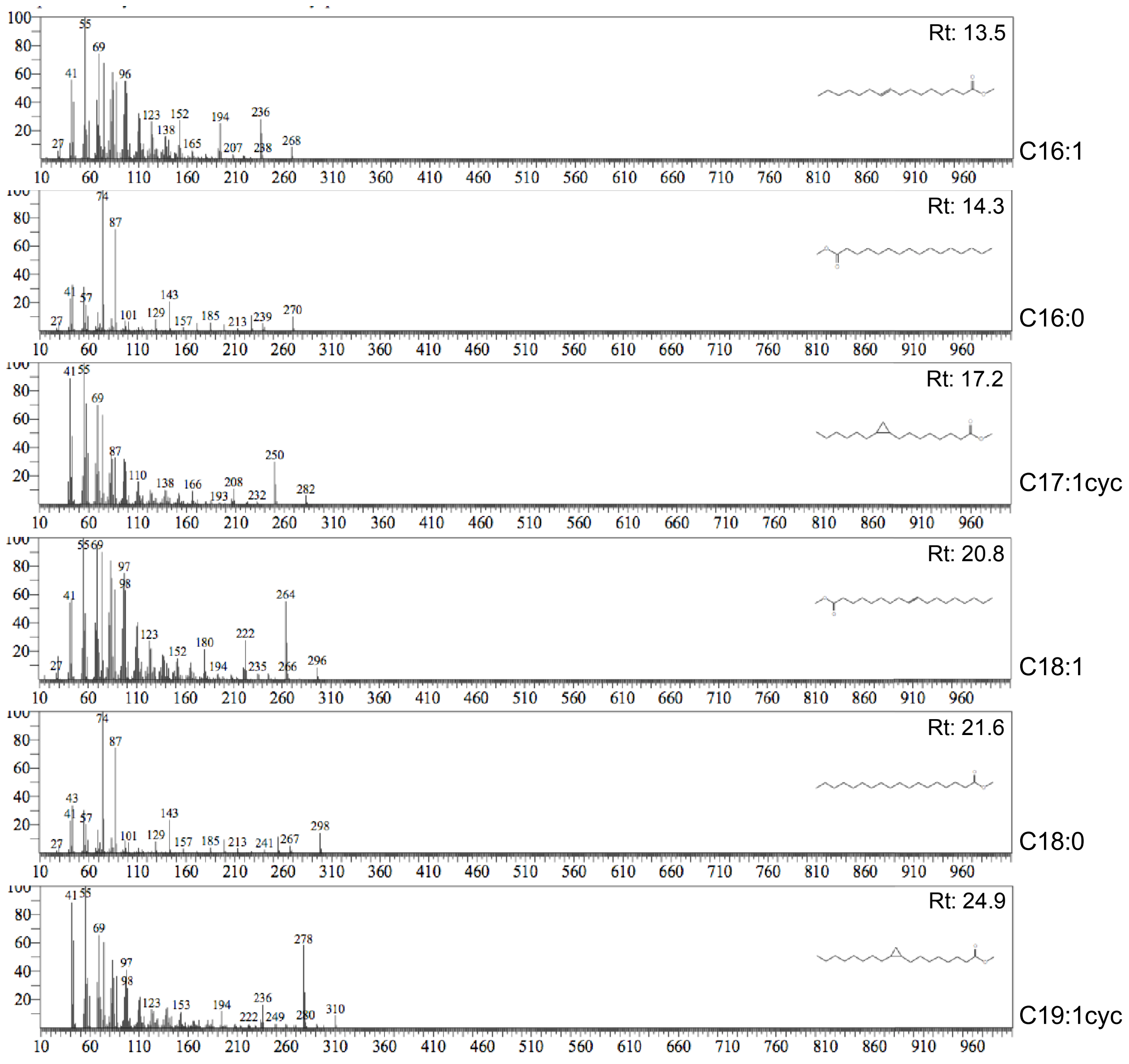

Supplementary Figure S3. Construction of desA and desB deletion alleles in various strain backgrounds. (A) Schematic map of desA (A) and desB (B) deletion cassette. (C) PCR assay using primer pair F1-R1. Because of the presence of the fabA rescue plasmid, chromosomal copy (no lable) and plasmid copy (labled with pl) are indicated. PCR fragment derived from deletion allele is smaller than that of wild type.

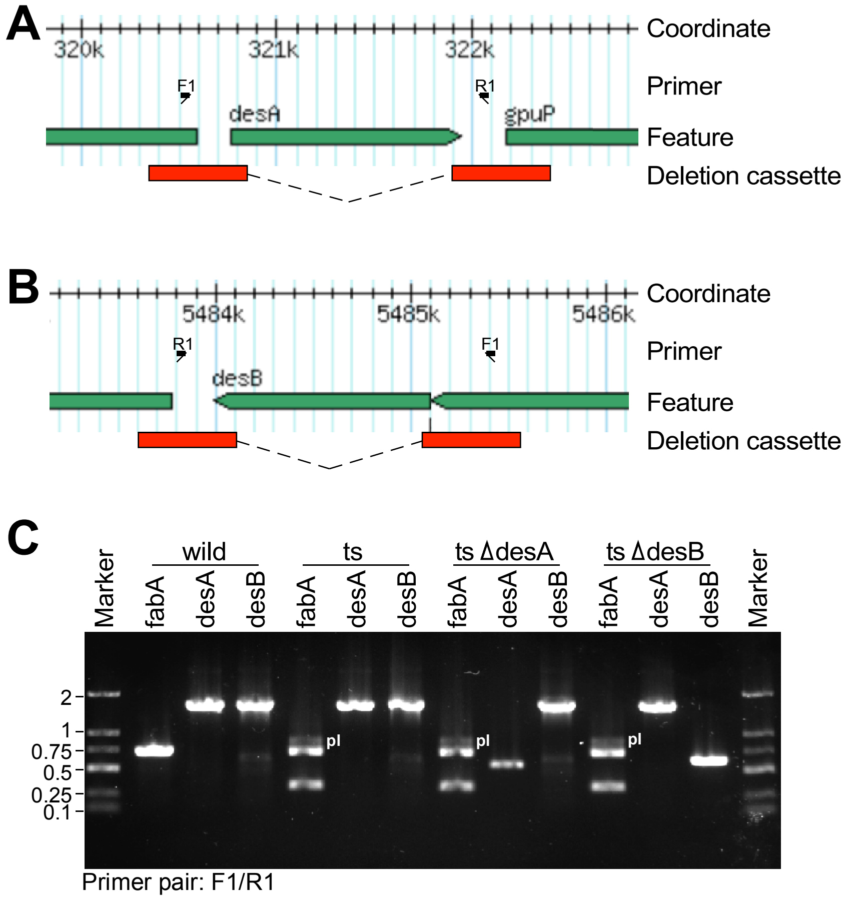

Supplementary table S1. Relative levels of fatty acid species based on GC-MS analysis of FAME
Note: Strain and temperature are indicated; individual level, average and standard deviation are shown.

RT (min)	FA	wt-30C_1	wt-30C_2	wt-30C_3	wt-30C_ave	wt-30C_SD
13.5	C16:1	10.46	10.72	10.1	10.43	0.31
14.3	C16:0	31.6	32.1	31.2	31.63	0.45
17.2	C17:1cyc	2.73	2.58	2.81	2.71	0.12
20.8	C18:1	36.7	34.3	38.2	36.40	1.97
21.6	C18:0	2.91	2.75	2.97	2.88	0.11
24.9	C19:1cyc	5.6	5.75	5.42	5.59	0.17

RT (min)	FA	wt, 42C_1	wt, 42C_2	wt, 42C_3	wt-42C_ave	wt-42C_SD
13.5	C16:1	3.82	3.9	4.05	3.92	0.12
14.3	C16:0	36.5	38.6	36.8	37.30	1.14
17.2	C17:1cyc	4.33	4.52	4.65	4.50	0.16
20.8	C18:1	21.9	22.4	22.8	22.37	0.45
21.6	C18:0	3.4	3.25	3.5	3.38	0.13
24.9	C19:1cyc	16.9	18.8	19.4	18.37	1.31

RT (min)	FA	ts_30C_1	ts_30C_2	ts_30C_3	ts-30C_ave	ts-30C_SD
13.5	C16:1	11.5	13.1	14.6	13.07	1.55
14.3	C16:0	30.6	32.6	34	32.40	1.71
17.2	C17:1cyc	2	2.15	2.2	2.12	0.10
20.8	C18:1	31	32.8	34.5	32.77	1.75
21.6	C18:0	4.8	5.1	5.35	5.08	0.28
24.9	C19:1cyc	4.1	4.4	4.7	4.40	0.30

RT (min)	FA	ts_42C_1	ts_42C_2	ts_42C_3	ts-42C_ave	ts-42C_SD
13.5	C16:1	2.15	2.3	2.4	2.28	0.13
14.3	C16:0	38.6	40	41.5	40.03	1.45
17.2	C17:1cyc	3.76	3.9	3.9	3.85	0.08
20.8	C18:1	17	18.7	19.5	18.40	1.28
21.6	C18:0	8.1	8.2	8.2	8.17	0.06
24.9	C19:1cyc	15.8	17	18	16.93	1.10

Supplementary table S2. Relative levels of fatty acid species in sup
Note: ND stands for not detected. Average and Standard deviation are shown.

RT (min)	FA	sup-30C_1	sup-30C_2	sup-30C_3	sup-30C_ave	sup-30C_SD
13.5	C16:1	14.6	14.4	13.5	14.17	0.59
14.3	C16:0	51	50.8	55.2	52.33	2.48
20.8	C18:1	ND	ND	ND		
21.6	C18:0	22.6	22.8	24.9	23.43	1.27

RT (min)	FA	sup-42C_1	sup-42C_2	sup-42C_3	sup-42C_ave	sup-42C_SD
13.5	C16:1	8.1	8.6	8.8	8.50	0.36
14.3	C16:0	48.3	48	44.9	47.07	1.88
20.8	C18:1	ND	ND	ND		
21.6	C18:0	33.5	33.8	36.1	34.47	1.42

lementary table S3. Small indel and SNP analysis in sup and fabA(Ts)

NP loci specific to sup, not present in fabA(Ts).

sup											fabA(Ts)										
POS_sup	REF	ALT	alt-co	all-co	alt-pre	Gene_l	Gene_ID	SNP_Typ	Codon_char	function	POS_fabA	REF	ALT	alt-ce	all-co	alt-pr	Gene_lo	Gene_ID	SNP_Typ	Codon_char	function
320702	C	T	231	231	1		PA0285(-) / PA0286(+)			Promoter	NA										
4448856	G	C	62	63	0.98		PA3969 (+) / PA3970(-)			Terminator	NA										
4869855	T	G	120	120	1	474	PA4341	nonsyn	GAA->GAC	probable transcriptional	NA										
5336153	G	A	180	180	1	1549	PA4751	nonsyn	CCG->TCG	cell division protein FtsH	NA										
5676870	G	T	165	168	0.98	989	PA5040	nonsyn	GCG->GAG	Type 4 fimbrial biogenesi	NA										

NP loci in both sup and fabA(Ts).

sup											fabA(Ts)										
POS_sup	REF	ALT	alt-co	all-co	alt-pre	Gene_l	Gene_ID	SNP_Typ	Codon_char	function	POS_fabA	REF	ALT	alt-co	all-co	alt-pr	Gene_lo	Gene_ID	SNP_Typ	Codon_char	function
183697	T	G	201	201	1	930	PA0159	nonsyn	TGT->TGG	probable transcriptiona	183697	T	G	208	209	1	930	PA0159	nonsyn	TGT->TGG	probable transcriptiona
413850	T	C	93	93	1						413850	T	C	104	104	1					
721611	C	T	84	84	1						721611	C	T	83	84	0.99					
721622	C	T	80	80	1						721622	C	T	76	76	1					
721663	T	C	40	40	1						721663	T	C	52	52	1					
721664	C	T	38	38	1						721664	C	T	45	45	1					
721667	A	G	34	34	1						721667	A	G	40	40	1					
721670	A	G	32	32	1						721670	A	G	35	35	1					
721718	A	G	24	25	0.96						721718	A	G	26	26	1					
721725	C	T	22	23	0.96						721725	C	T	26	26	1					
721740	C	T	19	20	0.95						721740	C	T	22	22	1					
1589438	G	C	173	173	1	101	PA1459	nonsyn	GGC->GCC	probable methyltransfe	1589438	G	C	192	192	1	101	PA1459	nonsyn	GGC->GCC	probable methyltransf
2239547	T	G	93	93	1						2239547	T	G	115	115	1					
2669175	G	C	116	116	1	2455	PA2400	nonsyn	CCG->GCG	PvdJ	2669175	G	C	152	152	1	2455	PA2400	nonsyn	CCG->GCG	PvdJ
2807982	T	A	91	91	1	514	PA2492	nonsyn	TTC->ATC	transcriptional regulatc	2807982	T	A	130	131	0.99	514	PA2492	nonsyn	TTC->ATC	transcriptional regulato
4212201	A	G	159	159	1	1907	PA3760	nonsyn	CAC->CGC	N-Acetyl-D-Glucosamin	4212201	A	G	151	151	1	1907	PA3760	nonsyn	CAC->CGC	N-Acetyl-D-Glucosamir
4344266	A	G	178	178	1	570	PA3877	syn	CTT->CTC	nitrite extrusion protei	4344266	A	G	202	202	1	570	PA3877	syn	CTT->CTC	nitrite extrusion protei
4448855	C	G	71	72	0.99						4869855	T	G	122	122	1	474	PA4341	nonsyn	GAA->GAC	probable transcriptiona
4924552	C	G	153	153	1	532	PA4394	nonsyn	GTG->CTG	conserved hypothetical	4924552	C	G	160	160	1	532	PA4394	nonsyn	GTG->CTG	conserved hypothetica
4924553	G	C	152	152	1	531	PA4394	syn	CCC->CCG	conserved hypothetical	4924553	G	C	158	158	1	531	PA4394	syn	CCC->CCG	conserved hypothetica
5036891	A	C	170	170	1						5036891	A	C	238	238	1					
5743461	C	G	146	146	1	1293	PA5100	syn	ACG->ACC	urocanase	5743461	C	G	168	168	1	1293	PA5100	syn	ACG->ACC	urocanase
5743462	G	C	145	145	1	1292	PA5100	nonsyn	ACG->AGG	urocanase	5743462	G	C	170	170	1	1292	PA5100	nonsyn	ACG->AGG	urocanase
6079222	A	G	165	165	1	1179	PA5399	syn	CTA->CTG	DgcB, Dimethylglycine	6079222	A	G	152	152	1	1179	PA5399	syn	CTA->CTG	DgcB, Dimethylglycine
6098781	G	C	183	184	0.99	1758	PA5418	syn	GGG->GGC	sarcosine oxidase alpha	6098781	G	C	169	169	1	1758	PA5418	syn	GGG->GGC	sarcosine oxidase alph
6115455	T	G	201	201	1	858	PA5434	nonsyn	AAA->AAC	tryptophan permease	6115455	T	G	228	228	1	858	PA5434	nonsyn	AAA->AAC	tryptophan permease

ndel loci in both sup and fabA(Ts).

sup

POS_sup	REF	ALT	alt-cov	all-cov	alt-pre	Gene_l	Gene_ID	Indel_ty	Mut_type	fucntion
169283	CG	C	199	199	1			Del		
411125	AC	A	157	165	0.95			Del		
667028	G	GC	173	173	1			Ins		
740419	G	GC	174	174	1	217	PA0683	Ins	Frame chan	probable type II secreti
816529	G	GC	137	137	1			Ins		
891099	A	AC	150	153	0.98			Ins		
1116213	G	GC	231	231	1	1	PA1029	Ins	Frame chan	hypothetical protein
1215657	A	AG	155	156	0.99	374	PA1122	Ins	Frame chan	probable peptide defor
1275766	GA	G	173	173	1	33	PA1174	Del	Frame chan	periplasmic nitrate red
1440622	CA	C	139	139	1	1917	PA1327	Del	Frame chan	probable protease
1445357	A	AG	116	116	1			Ins		
1467482	A	AG	101	101	1			Ins		
1467483	C	CG	102	102	1			Ins		
1491398	GCC	G	164	164	1	207	PA1374	Del	Frame chan	hypothetical protein
1835045	G	GC	123	123	1	653	PA1685	Ins	Frame chan	enolase-phosphatase E
2169348	A	AG	112	115	0.97			Ins		
2186927	G	GC	26	32	0.81			Ins		
2195457	G	GC	86	88	0.98			Ins		
2239555	A	AG	119	119	1			Ins		
2342110	C	CT	193	194	0.99			Ins		
2355771	A	AG	139	139	1	88	PA2139	Ins	Frame chan	hypothetical protein
2356681	GC	G	129	129	1	514	PA2141	Del	Frame chan	hypothetical protein
2532046	G	GC	124	124	1			Ins		
2753522	G	GC	151	151	1	924	PA2452	Ins	Frame chan	hypothetical protein
2807693	TCGC	T	138	140	0.99	225	PA2492	Del	Frame chan	transcriptional regulatc
3016844	G	GC	175	175	1	41	PA2668	Ins	Frame chan	hypothetical protein
3083196	A	AG	154	154	1	3347	PA2727	Ins	Frame chan	hypothetical protein
3919508	G	GC	191	191	1			Ins		
4539468	G	GC	112	113	0.99	313	PA4059	Ins	Frame chan	hypothetical protein
4888194	A	AG	174	174	1	2	PA4360	Ins	Frame chan	hypothetical protein
5033101	G	GC	112	112	1	1614	PA4496	Ins	Frame chan	probable binding prote
5071543	AACT	A	204	204	1			Del		
5472415	C	CG	193	193	1			Ins		
5655220	C	CCGC	141	142	0.99	410	PA5024	Ins	Ins	conserved hypothetical

fabA

POS_fabA(	REF	ALT	alt-co	all-co	alt-pr	Gene_l	Gene_ID	Indel_ty	Mut_type	fucntion
169283	CG	C	180	180	1			Del		
411125	AC	A	147	151	0.97			Del		
667028	G	GC	153	153	1			Ins		
740419	G	GC	142	142	1	217	PA0683	Ins	Frame chan	probable type II secret
816529	G	GC	119	119	1			Ins		
891099	A	AC	135	137	0.99			Ins		
1116213	G	GC	174	174	1	1	PA1029	Ins	Frame chan	hypothetical protein
1215657	A	AG	113	114	0.99	374	PA1122	Ins	Frame chan	probable peptide defo
1275766	GA	G	145	145	1	33	PA1174	Del	Frame chan	periplasmic nitrate red
1440622	CA	C	143	143	1	1917	PA1327	Del	Frame chan	probable protease
1445357	A	AG	92	92	1			Ins		
1467482	A	AG	83	83	1			Ins		
1467483	C	CG	83	83	1			Ins		
1491398	GCC	G	127	127	1	207	PA1374	Del	Frame chan	hypothetical protein
1835045	G	GC	97	97	1	653	PA1685	Ins	Frame chan	enolase-phosphatase E
2169348	A	AG	93	93	1			Ins		
2186927	G	GC	54	56	0.96			Ins		
2195457	G	GC	60	62	0.97			Ins		
2239555	A	AG	90	90	1			Ins		
2342110	C	CT	143	143	1			Ins		
2355771	A	AG	104	104	1	88	PA2139	Ins	Frame chan	hypothetical protein
2356681	GC	G	84	84	1	514	PA2141	Del	Frame chan	hypothetical protein
2532046	G	GC	108	108	1			Ins		
2753522	G	GC	123	123	1	924	PA2452	Ins	Frame chan	hypothetical protein
2807693	TCGC	T	106	108	0.98	225	PA2492	Del	Frame chan	transcriptional regulatc
3016844	G	GC	108	108	1	41	PA2668	Ins	Frame chan	hypothetical protein
3083196	A	AG	127	127	1	3347	PA2727	Ins	Frame chan	hypothetical protein
3919508	G	GC	140	140	1			Ins		
4539468	G	GC	106	106	1	313	PA4059	Ins	Frame chan	hypothetical protein
4888194	A	AG	126	126	1	2	PA4360	Ins	Frame chan	hypothetical protein
5033101	G	GC	90	90	1	1614	PA4496	Ins	Frame chan	probable binding prote
5071543	AACT	A	141	141	1			Del		
5472415	C	CG	154	154	1			Ins		
5655220	C	CCGC	103	105	0.98	410	PA5024	Ins	Ins	conserved hypothetica

Supplementary Table S4. Ratios between sup and wild type, sup and fabA(Ts) of 39 common DEGs.
Note, samples are in triplicate and all ratios are in log2 scale.

ID	NAME	sup/wt_3	sup/wt_3	sup/wt_3	sup/wt_4	sup/wt_4	sup/wt_4	sup/ts_3	sup/ts_3	sup/ts_3	sup/ts_4	sup/ts_4	sup/ts_42
PA0286	desA	1.838	1.807	1.801	1.743	1.723	1.748	1.265	1.234	1.227	0.905	0.885	0.910
PA0461	yihG	1.391	1.380	1.330	1.890	1.899	1.848	1.472	1.461	1.411	1.547	1.557	1.506
PA0485	rarD	1.556	1.595	1.649	3.787	3.848	3.817	2.464	2.503	2.557	2.800	2.861	2.830
PA0667	mepM	1.012	1.032	1.020	0.865	0.936	0.946	1.235	1.254	1.243	1.064	1.135	1.145
PA0729	pfiT	0.938	0.954	1.012	4.643	4.542	4.612	2.465	2.482	2.540	3.563	3.461	3.532
PA0730	conserve	1.833	1.843	1.859	4.218	4.211	4.198	2.897	2.907	2.923	2.904	2.896	2.884
PA0762	algU	1.194	1.184	1.185	5.294	5.286	5.271	1.640	1.630	1.631	4.261	4.253	4.237
PA0765	mucC	1.563	1.545	1.576	2.730	2.840	2.783	2.022	2.004	2.034	1.561	1.670	1.614
PA0783	putP	0.986	0.988	0.977	3.214	3.163	3.206	1.310	1.312	1.301	1.658	1.607	1.650
PA0942	conserve	1.468	1.359	1.413	3.034	3.128	3.084	2.705	2.596	2.650	2.549	2.642	2.598
PA1030	hypothet	2.415	2.453	2.442	4.601	4.535	4.559	2.910	2.947	2.936	3.421	3.355	3.379
PA1077	flgB	2.420	2.449	2.430	3.098	3.092	3.150	1.361	1.390	1.371	2.825	2.819	2.878
PA2003	bdhA	2.400	2.410	2.423	3.075	3.030	3.027	0.849	0.858	0.872	2.666	2.621	2.617
PA2318	hypothet	5.288	5.295	5.305	4.352	4.362	4.326	1.943	1.950	1.960	3.787	3.797	3.761
PA2662	conserve	1.385	1.398	1.400	4.052	4.007	4.065	4.480	4.493	4.494	2.412	2.368	2.425
PA2663	ppyR	1.400	1.430	1.336	5.069	5.098	5.102	3.776	3.806	3.712	3.493	3.521	3.526
PA2664	fhp	1.705	1.701	1.729	8.243	8.222	8.266	7.089	7.086	7.113	6.125	6.104	6.148
PA2737	conserve	0.913	0.938	0.890	2.549	2.560	2.532	1.455	1.481	1.432	1.142	1.153	1.126
PA2798	conserve	1.209	1.240	1.204	1.456	1.418	1.490	0.915	0.946	0.910	1.155	1.117	1.189
PA3007	lexA	2.466	2.446	2.475	4.356	4.364	4.372	3.622	3.602	3.631	3.143	3.150	3.159
PA3008	sulA	2.124	2.071	2.067	3.627	3.722	3.719	3.055	3.003	2.998	2.239	2.334	2.331
PA3009	hypothet	1.389	1.466	1.467	2.884	3.074	2.896	1.965	2.042	2.043	2.642	2.832	2.655
PA3142	conserve	1.191	1.067	1.104	3.131	3.148	3.111	1.465	1.341	1.377	2.362	2.379	2.343
PA3353	flgZ	1.038	1.055	1.027	1.624	1.680	1.589	1.322	1.339	1.311	1.485	1.542	1.451
PA3413	yebG	3.051	3.058	3.066	4.780	4.813	4.805	2.794	2.801	2.809	4.919	4.951	4.943
PA3617	recA	2.329	2.338	2.342	4.678	4.676	4.665	2.998	3.007	3.011	3.632	3.629	3.618
PA3662	hypothet	1.124	1.099	1.088	1.112	1.169	1.185	1.675	1.650	1.638	0.920	0.977	0.992
PA3732	yjfI	3.038	2.998	3.033	5.632	5.681	5.642	4.113	4.073	4.108	3.760	3.809	3.770
PA3743	trmD	1.344	1.364	1.360	3.971	3.953	3.953	3.080	3.099	3.096	3.419	3.401	3.401
PA3744	rimM	0.899	0.906	0.904	2.211	2.234	2.250	2.089	2.096	2.094	1.838	1.860	1.877
PA3756	yafK	1.409	1.319	1.365	1.814	1.794	1.813	1.646	1.556	1.602	1.388	1.368	1.387
PA4635	mgtC	1.064	1.029	1.087	3.820	3.757	3.847	1.894	1.858	1.916	2.780	2.717	2.807
PA4942	hflK	1.569	1.557	1.544	1.987	2.022	2.002	1.638	1.626	1.613	1.133	1.168	1.148
PA5012	waaF	1.578	1.558	1.564	1.955	1.927	1.974	1.527	1.506	1.513	1.375	1.347	1.394
PA5177	yrfG	0.855	0.906	0.902	1.915	1.947	1.862	0.946	0.997	0.993	1.023	1.055	0.971
PA5313	gabT2	1.033	1.043	0.988	2.038	1.942	2.002	1.780	1.790	1.735	1.271	1.175	1.234
PA5469	conserve	1.283	1.317	1.313	6.432	6.405	6.400	2.929	2.964	2.959	5.628	5.600	5.595
PA5524	conserve	1.093	1.130	1.058	2.136	2.133	2.129	2.146	2.183	2.111	1.098	1.095	1.091
PA5555	atpG	0.856	0.853	0.844	0.938	0.937	0.922	1.794	1.791	1.782	0.881	0.879	0.865
